# *Klebsiella pneumoniae* hijacks the Toll-IL-1R protein SARM1 in a type I IFN-dependent manner to antagonize host immunity

**DOI:** 10.1016/j.celrep.2022.111167

**Published:** 2022-08-09

**Authors:** Claudia Feriotti, Joana Sá-Pessoa, Ricardo Calderón-González, Lili Gu, Brenda Morris, Ryoichi Sugisawa, Jose L. Insua, Michael Carty, Amy Dumigan, Rebecca J. Ingram, Adrien Kissenpfening, Andrew G. Bowie, José A. Bengoechea

**Affiliations:** 1 Wellcome-Wolfson Institute for Experimental Medicine, School of Medicine, Dentistry and Biomedical Sciences, Queen’s University Belfast, 97 Lisburn Road, Belfast, UK; 2 School of Biochemistry and Immunology, Trinity Biomedical Sciences Institute, Trinity College Dublin, Dublin 2, Ireland

**Keywords:** SARM1, *Klebsiella pneumoniae*, AIM2 inflammasome, type I IFN

## Abstract

Many bacterial pathogens antagonize host defense responses by translocating effector proteins into cells. It remains an open question how those pathogens not encoding effectors counteract anti-bacterial immunity. Here, we show that *Klebsiella pneumoniae* exploits the evolutionary conserved innate protein SARM1 to regulate negatively MyD88- and TRIF-governed inflammation, and the activation of the MAP kinases ERK and JNK. SARM1 is required for *Klebsiella* induction of interleukin-10 (IL-10) by fine-tuning the p38-type I interferon (IFN) axis. SARM1 inhibits the activation of *Klebsiella*-induced absent in melanoma 2 inflammasome to limit IL-1β production, suppressing further inflammation. *Klebsiella* exploits type I IFNs to induce SARM1 in a capsule and lipopolysaccharide O-polysaccharide-dependent manner via the TLR4-TRAM-TRIF-IRF3-IFNAR1 pathway. Absence of SARM1 reduces the intracellular survival of *K. pneumoniae* in macrophages, whereas *sarm1*-deficient mice control the infection. Altogether, our results illustrate an anti-immunology strategy deployed by a human pathogen. SARM1 inhibition will show a beneficial effect to treat *Klebsiella* infections.

## Highlights

• SARM1 is an evolutionary conserved innate immune protein with a TIR domain
• *Klebsiella pneumoniae* induces SARM1 to limit inflammation, and to induce IL-10
• SARM1 inhibits the action of AIM2 inflammasome to limit IL-1β production
• *In vivo*, absence of SARM1 facilitates the clearance of *Klebsiella pneumoniae*

## Graphical abstract

**Figure:**
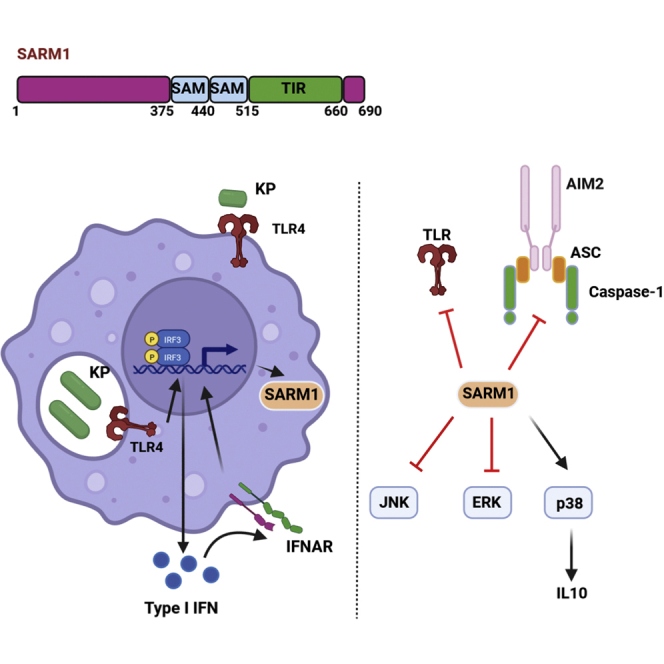

## Teaser

Feriotti et al. uncover how *Klebsiella pneumoniae* exploits the conserved innate immune protein SARM1 to limit inflammation and to induce IL-10. SARM1 also inhibits *K. pneumoniae*-induced AIM2 inflammasome. *In vivo*, absence of SARM1 facilitates the clearance of the pathogen, revealing an Achilles heel of our immune system exploited by *Klebsiella.*

## Introduction

*Klebsiella pneumoniae* (KP) is one of the pathogens sweeping the world in the antimicrobial resistance pandemic. More than a third of KP isolates reported to the European Center for Disease Prevention and Control were resistant to at least one antimicrobial group; the most common resistance phenotype being combined resistance to fluoroquinolones, third-generation cephalosporins, and aminoglycosides (Penalva et al., 2019). KP infections are associated with high morbidity and mortality (Giske et al., 2008), and there is an increase in the number of community-acquired infections worldwide (Lipworth et al., 2021; Magiorakos et al., 2013). Recent studies have recognized that KP strains have access to a mobile pool of virulence genes (Holt et al., 2015; Lam et al., 2018), enabling the emergence of a multidrug, hypervirulent KP clone capable of causing untreatable infections in healthy individuals. Worryingly, there are reports describing such strains (Gu et al., 2018; Yao et al., 2018; Zhang et al., 2015, 2016).

An attractive approach to develop new therapeutics against KP infections is to boost innate defences (Bengoechea and Sa Pessoa, 2019). This pathway requires an in-depth understanding of the strategies used by KP to survive within the infected tissue and of how KP controls the activation of immune cells. Of note, KP does not encode any type III and IV secretion systems known to deploy proteins to subvert immune cells.

Successful elimination of infections by the innate immune system is dependent on the activation of pattern recognition receptors (PRRs). The PRRs Toll-like receptor (TLR) 4 and TLR2 restrict KP infection (Wieland et al., 2011). Both receptors signal via the adaptors MyD88 and TRIF to activate NF-κB and IRF3, respectively. These transcription factors and MAP kinases control the activation of antimicrobial responses (Jenner and Young, 2005). The fact that *IL1R*^*−/−*^ mice are susceptible to KP infection demonstrates the importance of interleukin-1β (IL-1β)-controlled responses for bacterial clearance (Cai et al., 2012). Production of the mature active form of IL-1β requires the expression of the pro-IL-1β, following PRR-mediated recognition of a pathogen, and its cleavage by caspase-1 to release the active form of the cytokine. The activation of caspase-1 also leads to a form of cell death termed pyroptosis through the proteolytic cleavage of gasdermin-D (GSDMD). The activation of caspase-1 requires the assembly of the inflammasome multiprotein platform. Evidence suggests that KP induces the secretion of IL-1β *in vivo* and *in vitro* via inflammasome activation (Cai et al., 2012; Willingham et al., 2009). Whether KP has evolved any strategy to limit early events of TLR signaling and inflammasome activation remains an open question.

SARM1 (sterile α and HEAT armadillo motif-containing protein) is an evolutionary conserved innate immune protein across mammalian species with identities higher than 90% (Belinda et al., 2008). Analysis of human SARM1 revealed a selective pressure to preserve the integrity of the protein (Fornarino et al., 2011). SARM1 contains a Toll-IL-1R (TIR) domain (Bratkowski et al., 2020; O'Neill and Bowie, 2007). The presence of this domain indicates a role in IL1 and TLR signaling. Interestingly, bacterial proteins containing the TIR domain interfere with TLR signaling to inhibit innate responses (Askarian et al., 2014; Cirl et al., 2008; Coronas-Serna et al., 2020; Imbert et al., 2017; Xiong et al., 2019). It is intriguing that the SARM1 TIR domain is more closely related to bacteria TIR proteins than to the other mammalian TIR containing adaptors (Zhang et al., 2011). Therefore, SARM1 may play a negative role regulating TLR signaling. Indeed, there are data suggesting that SARM1 inhibits lipopolysaccharide (LPS)-induced signaling via TLR4-TRIF and MyD88 pathways (Carlsson et al., 2016; Carty et al., 2006). Recent work uncovered SARM1-negative regulation of the NLRP3 inflammasome (Carty et al., 2019). Collectively, this evidence led us to speculate whether KP may hijack SARM1, an endogenous TIR-containing protein regulating TLR and inflammasome activation, to control immune responses. The role of SARM1 in infections has been conclusively established to restrict West Nile virus infection (Szretter et al., 2009; Uccellini et al., 2020). However, and to the best of our knowledge, there is no evidence supporting any role of SARM1 in bacterial infections.

Here, we reveal KP leverages the immunomodulatory roles of SARM1 to control cell intrinsic immunity. We show KP negatively regulates TLR-governed inflammatory responses via SARM1. We demonstrate that SARM1 is required for KP induction of IL-10. We identify absent in melanoma 2 (AIM2) as the inflammasome activated by KP inhibited by SARM1 to limit IL-1β production. We establish KP exploits type I IFNs to induce SARM1 in a capsule (CPS) and LPS O-polysaccharide-dependent manner. *In vitro*, absence of SARM1 reduces the intracellular survival of KP whereas, *in vivo*, *sarm1*^*−/−*^ mice clear the infection. Our findings illustrate the crucial role of SARM1 in KP immune evasion strategies, revealing one of the Achilles heels of our immune system exploited by the pathogen to overcome host protective responses.

## Results

### SARM1 negatively regulates KP-induced inflammation

**Figure 1:**
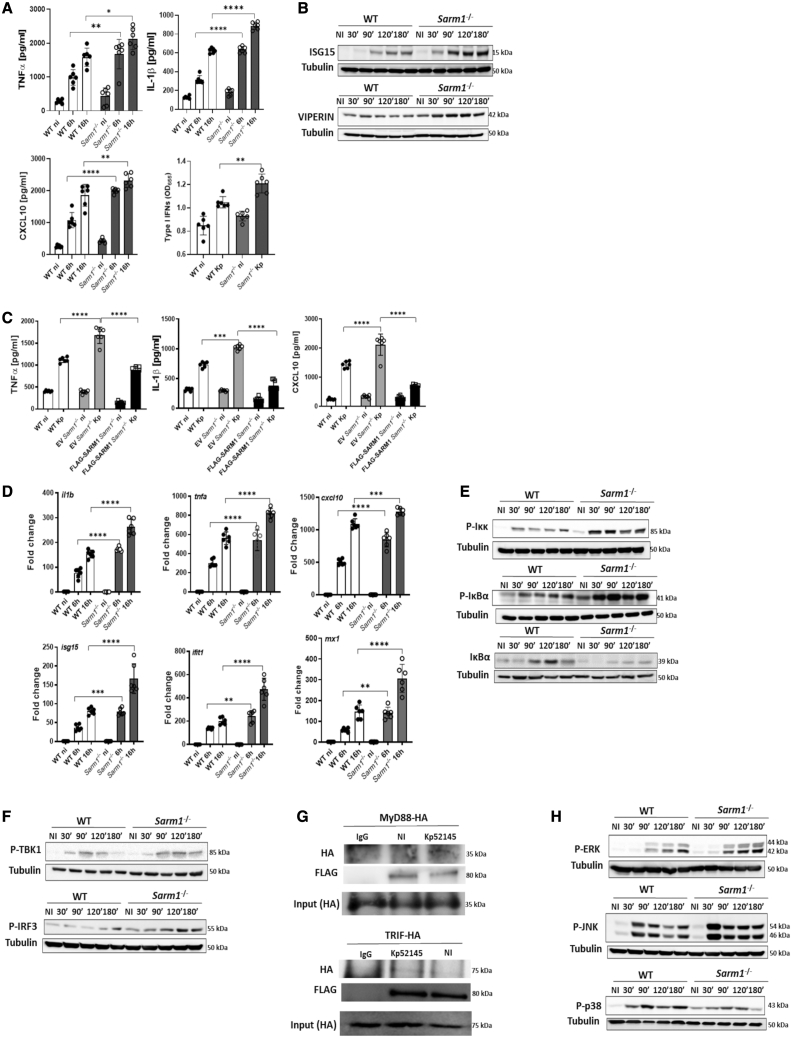
SARM1 negatively regulates *K. pneumoniae*-induced inflammation (A) ELISA of TNF-α, IL-1β, and CXCL10 secreted by infected macrophages for 6 and 16 h. Type I IFN levels determined in the supernatants of macrophages 16 h post infection. (B) Immunoblot analysis of ISG15 and Viperin in infected cells. (C) ELISA of TNF-α, IL-1β, and CXCL10 secreted by wild-type (WT) macrophages, and retrovirally transfected *sarm1*^*−/−*^ cells with FLAG-SARM1 or control vector (EV) 16 h post infection. (D) *il1b*, *tnfa*, *cxcl10*, *isg15*, *ifit1*, and *mx1* mRNA levels in infected macrophages for 6 and 16 h. (E and F) Immunoblot analysis of phosphorylated Iκκα/β (P-Iκκ), phosphorylated IκBα (P-IκBα), total IκBα (IκBα) (E), phosphorylated TBK1 (P-TBK1), and phosphorylated Irf3 (P-IRF3) (F) in infected cells. (G) *Sarm1*^*FLAG*^ macrophages were transfected with MyD88-HA or TRIF-HA plasmids, and the following day infected with Kp52145. Lysates were immunoprecipitated using anti-FLAG antibody. Preimmune mouse IgG served as negative control. MyD88-HA and TRIF-HA levels in the input lysates before immunoprecipitation are shown. (H) Immunoblot analysis of phosphorylated ERK (P-ERK), phosphorylated JNK (P-JNK), phosphorylated p38 (P-p38), and tubulin levels in lysates of infected cells. In (B) and (E–H) images are representative of three independent experiments. In (A), (C), and (D) values are presented as the mean ± SD of three independent experiments measured in duplicate. ^∗∗∗∗^p ≤ 0.0001, ^∗∗∗^p ≤ 0.001, ^∗∗^p ≤ 0.01, ^∗^p ≤ 0.05 for the indicated comparisons determined using one way-ANOVA with Bonferroni contrast for multiple comparisons test.

To examine the effect of SARM1 on KP-induced responses, we infected immortalized bone marrow-derived macrophages (iBMDMs) from wild-type and *sarm1*^*−/−*^ mice with the KP hypervirulent strain CIP52.145 (hereafter Kp52145). This strain belongs to the KpI group and it encodes all virulence functions associated with invasive community-acquired disease in humans (Holt et al., 2015; Lery et al., 2014). Infected *sarm1*^*−/−*^ cells secreted higher levels of the MyD88-dependent cytokines tumor necrosis factor α (TNF-α), and IL-1β, and of the TRIF-dependent cytokines CXCL10 and type I IFNs than infected wild-type macrophages (Figure 1A). The levels of the TRIF-dependent proteins ISG15 and Viperin were also higher in the lysates of Kp52145-infected *sarm1*^*−/−*^ macrophages than in those of wild-type cells (Figure 1B). Two strains of the carbapenem-resistant KP sequence type 258 (ST258), KP35, and NJST258_2 (Ahn et al., 2016; Deleo et al., 2014), induced higher levels of TNF-α, IL-1β, CXCL10, and type I IFNs in *sarm1*^*−/−*^ macrophages than in wild-type cells (Figure S1), demonstrating that SARM1 contribution to KP-triggered inflammatory responses is observed following infection with multidrug resistant strains. Kp52145 also increased the levels of the MyD88-dependent cytokines TNF-α, and IL-1β, and of the TRIF-dependent cytokine CXCL10 in *sarm1*^*−/−*^ BMDMs (Figure S2A), ruling out that the heightened responses observed in inmortalized cells were caused by the immortalization protocol. Rescue experiments by retroviral expression of FLAG-SARM1 in *sarm1*^*−/−*^ iBMDMs confirmed that the phenotype was due to the absence of the SARM1 protein because the levels of TNF-α, IL-1β, and CXCL10 in FLAG SARM1 were lower than those found in infected *sarm1*^*−/−*^ cells (Figure 1C). Collectively, these data demonstrate that SARM1 negatively regulates KP-induced inflammation.

Commercially available *sarm1*^*−/−*^ mice carry a passenger mutation that may affect cytokine responses (Uccellini et al., 2020). Although the cytokines affected are not those assessed in our study, we examined the role of SARM1 in KP infection by reducing its levels via siRNA. Control experiments confirmed the reduction of the *sarm1* transcript in iBMDMs (Figure S2B). We again found higher levels of IL-1β, TNF-α, and CXCL10 in the supernatants of infected *sarm1* knockdown macrophages than in macrophages transfected with a non-targeting (All Stars) siRNA control (Figure S2C). To provide additional support to our observations, we challenged iBMDMs from a new knockout SARM1 strain generated using CRISPR-Cas9-mediated genome engineering, *Sarm1*^*em1.1Tf*^ (Doran et al., 2021). Kp52145 induced a heightened inflammatory response in *Sarm1*^*em1.1Tft*^ macrophages compared with wild-type ones from littermates (Figure S2D). Altogether, these data provide further evidence supporting the role of SARM1 to attenuate KP-induced inflammation.

We next investigated whether the absence of SARM1 affects the transcription of MyD88- and TRIF-dependent cytokines. Figure 1D shows that Kp52145 increased the transcription of the MyD88-governed cytokines *tnfa* and *il1b*, and of the TRIF-controlled cytokine *ifnb* in *sarm1*^*−/−*^ macrophages compared with wild-type ones. The transcription of the interferon-stimulated genes (ISG) *isg15*, *mx1*, and *ifit1* was also upregulated in infected *sarm1*^*−/−*^ cells (Figure 1D).

Because NF-κB and IRF3 govern MyD88- and TRIF-dependent responses, respectively, we next investigated whether SARM1 regulates these pathways in KP-infected cells. The IKKα/β kinase controls the phosphorylation of IκBα that leads to the subsequent degradation of the protein by the ubiquitin proteasome, allowing the nuclear translocation of NF-κB (Taniguchi and Karin, 2018). Immunoblotting analysis showed an increase in the phosphorylation of IKKα/β in infected *sarm1*^*−/−*^ macrophages (Figure 1E). We also observed an increased phosphorylation of IκBα with a concomitant reduction in the levels of total IκBα in Kp52145-infected *sarm1*^*−/−*^ macrophages compared with wild-type ones (Figure 1E). Altogether, these results show an enhance activation of NF-κB in infected *sarm1*^*−/−*^ macrophages. To investigate the activation of the IRF3 signaling cascade, we assessed the phosphorylation of TBK1 and IRF3. TBK1 is the kinase phosphorylating IRF3, which it is an essential event for its nuclear translocation (Fitzgerald et al., 2003). Immunoblotting experiments revealed an increase phosphorylation of TBK1 and IRF3 in Kp52145-infected *sarm1*^*−/−*^ macrophages (Figure 1F), confirming an increase activation of IRF3 in the absence of SARM1.

Reconstitution experiments in HEK293 cells by transfecting SARM1, and either MyD88 or TRIF, and reporter systems to assess activation of NF-κB and IRF3 demonstrated that SARM1 interacts with MyD88 and TRIF to block the activation of these signaling pathways (Carlsson et al., 2016; Carty et al., 2006). Therefore, we sought to determine whether KP infection would induce the interaction between SARM1 and MyD88 or SARM1 and TRIF. There is no commercially available antibody to assess mouse SARM1 protein levels reliably. To facilitate these experiments, we took advantage of a recently described mouse expressing an epitope-tagged SARM1 endogenously with a triple FLAG tag and double strep tag on the C-terminal end, *Sarm1*^*FLAG*^ (Doran et al., 2021). Control experiments confirmed that the tagged proteins retain functionality (Doran et al., 2021). Figure 1G shows that in *Sarm1*^*FLAG*^ iBMDMs SARM1-FLAG co-immunoprecipitates MyD88-HA and TRIF-HA only in Kp52145-infected cells, indicating that KP-induced interaction of SARM1 with MyD88 and TRIF explains the reduced activation of NF-κB and IRF3.

We next assessed the activation of MAPKs due to their role in governing the expression of inflammatory genes (Dong et al., 2002). Indirect evidence suggests that SARM1 inhibits MAPK activation (Peng et al., 2010). Western blotting showed an increase in the levels of phosphorylated ERK and JNK in infected *sarm1*^*−/−*^ macrophages compared with infected wild-type cells (Figure 1H). In contrast, there was a reduction in the phosphorylation of p38 in infected *sarm1*^*−/−*^ macrophages (Figure 1H). Densitometry analysis of the images confirmed these results (Figure S3). These findings indicate that SARM1 exerts a negative effect on the activation of ERK and JNK, whereas SARM1 is needed for p38 activation upon KP infection.

### SARM1 is required for KP induction of IL-10 via p38

**Figure 2:**
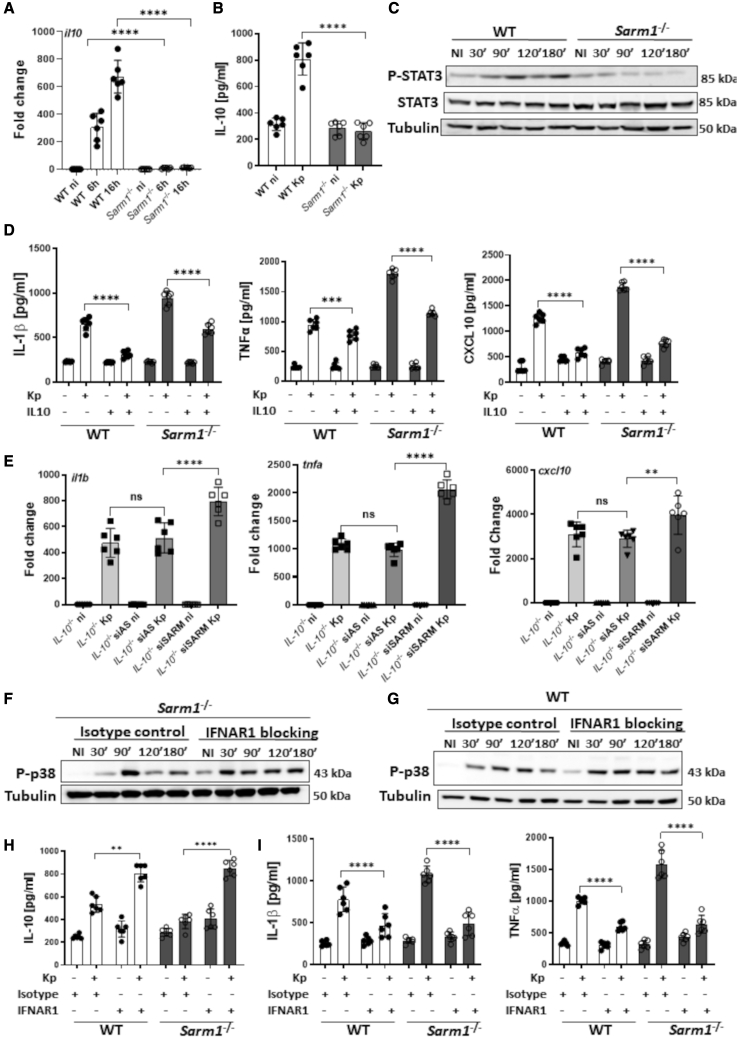
SARM1 is required for *K. pneumoniae* induction of IL-10 via p38 (A) *il10* mRNA levels in infected cells for 6 and 16 h. (B) ELISA of IL-10 secreted by infected cells for 16 h. (C) Immunoblot analysis of phosphorylated STAT3 (P-STAT3) and total STAT3 (STAT3) in infected cells. (D) ELISA of TNF-α, IL-1β, and CXCL10 secreted by infected cells for 16 h. Where indicated, cells were treated with recombinant IL-10 (1 ng/mL) overnight before infection. (E) *il1b*, *tnfa*, and *cxcl10* mRNA levels in *il10*^*−/−*^ macrophages, and *il10*^*−/−*^ cells transfected with All Stars siRNA control (AS), or SARM1 siRNA (siSARM) non-infected (ni) or infected (Kp) for 16 h. (F and G) Immunoblot analysis of phosphorylated p38 (P-p38) in *sarm1*^*−/−*^-infected (F) and WT-infected (G) cells Where indicated, cells were treated with isotype control antibody, or IFNAR1 blocking overnight before infection. (H) ELISA of IL-10, secreted by WT and *sarm1*^*−/−*^ macrophages non-infected or infected with Kp52145 for 16 h. Where indicated, cells were treated with isotype control antibody, or IFNAR1 blocking overnight before infection. (I) ELISA of IL-1β and TNF-α secreted by WT and *sarm1*^*−/−*^ macrophages non-infected or infected with Kp52145 for 16 h. Where indicated, cells were treated with isotype control antibody, or IFNAR1 blocking overnight before infection. In (C), (F), and (G) images are representative of three independent experiments. In (A), (B), (D), (E), (H), and (I) values are presented as the mean ± SD of three independent experiments measured in duplicate. ^∗∗∗∗^p ≤ 0.0001, ^∗∗∗^p ≤ 0.001, ^∗∗^p ≤ 0.01; ns, p > 0.05 for the indicated comparisons determined using one way-ANOVA with Bonferroni contrast for multiple comparisons test.

We next sought to ascertain the effect of the reduced p38 activation in the absence of SARM1. Because p38 activation is linked to the production of IL-10 (Saraiva and O'Garra, 2010), we asked whether SARM1 would affect KP induction of IL-10. The p38 inhibitor SB203580 abrogated Kp52145-induced production of IL-10 by wild-type cells (Figure S4A), connecting p38 activation and IL-10 production in KP-infected macrophages. *il10* transcription was decreased in the absence of SARM1 (Figure 2A), and the levels of IL-10 were lower in the supernatants of Kp52145-infected *sarm1*^*−/−*^ macrophages than in those from infected wild-type cells (Figure 2B). The reduced levels of IL-10 were consistent with the reduced phosphorylation of the IL-10-governed transcription factor STAT3 in Kp52145-infected *sarm1*^*−/−*^ macrophages (Figure 2C). The addition of recombinant IL-10 to Kp52145-infected *sarm1*^*−/−*^ macrophages decreased the levels of IL-1β, TNF-α, and CXCL10 (Figure 2D), suggesting that the reduced levels of IL-10 in the absence of SARM1 contributes to the upregulation of inflammation in infected *sarm1*^*−/−*^ macrophages. Kp52145 increased the levels of *il1b*, *tnfa*, and *cxcl10* in *il10*^*−/−*^ *sarm1* knockdown macrophages beyond the levels found in *il10*^*−/−*^-infected cells (Figure 2E), suggesting that the regulatory effect of SARM1 on inflammation is the sum of the IL-10-dependent attenuation, and the previously shown direct negative effect of SARM1 on MyD88 and TRIF. The efficiency of *sarm1* knockdown in the *il10*^−/−^ background is shown in Figure S4B.

To explain how the absence of SARM1 reduces the activation of p38, we reasoned that the heightened inflammation upon infection of *sarm1*^*−/−*^ macrophages might have a negative effect on p38 activation. Because there are reports demonstrating a connection between type I IFN signaling and p38 (Ivashkiv and Donlin, 2014), we speculated that the elevated levels of type I IFNs found in Kp52145-infected *sarm1*^*−/−*^ macrophages might underline the reduced p38 activation. Type I IFN production in KP-infected macrophages was detected as early as 30 min post infection using as readout the expression of the ISG *ifit1* (Figure S4C). This is consistent with the phosphorylation of TBK1 and IRF3 within 30–60 min post infection following the activation of TLR4-TRAM-TRIF signaling (Ivin et al., 2017). We then asked whether abrogating type I IFN signaling in the absence of SARM1 could rescue p38 activation. When the infection of *sarm1*^*−/−*^ macrophages was done in the presence of blocking antibodies against the type I IFN receptor (IFNAR1) there was an increase in the levels of phosphorylated p38 (Figure 2F). Likewise, we observed an increase in the levels of phosphorylated p38 in infected wild-type cells treated with the IFNAR1 receptor-blocking antibody (Figure 2G), reinforcing the connection between type I IFN levels and KP*-*induced p38 activation. As anticipated, the levels of IL-10 were higher in cells treated with the IFNAR1-blocking antibody than in those treated with the isotype control antibody (Figure 2H). In turn, we found a reduction in the levels of IL-1β, and TNF-α in the supernatants of cells treated with the blocking antibody (Figure 2I). The connection between type I IFN and p38 activation in KP-infected macrophages was further corroborated by the fact that Kp52145-induced p38 phosphorylation was higher in *ifnar1*^*−/−*^ cells than in wild-type ones (Figure S4D). Likewise, we found an increase phosphorylation of p38 in infected *tlr4*^*−/−*^ (Figure S4E) and *tram*^*−/−*^*trif*^*−/−*^ macrophages (Figure S4F), which is consistent with TLR4-TRAM-TRIF signaling mediating the production of KP-induced type I IFN (Ivin et al., 2017). As we anticipated, the levels of *il10* were higher in infected *tlr4*^*−/−*^, *tram*^*−/−*^*trif*^*−/−*^, and *ifnar1*^*−/−*^ macrophages compared with infected wild-type cells (Figure S4G).

Altogether, these data demonstrate that absence of SARM1 impairs KP-mediated p38 activation due to the negative regulation exerted by type I IFN. The reduced p38 activation limits the levels of IL-10 with a concomitant increase in inflammation.

### SARM1 negatively regulates KP-induced AIM2 inflammasome activation

**Figure 3:**
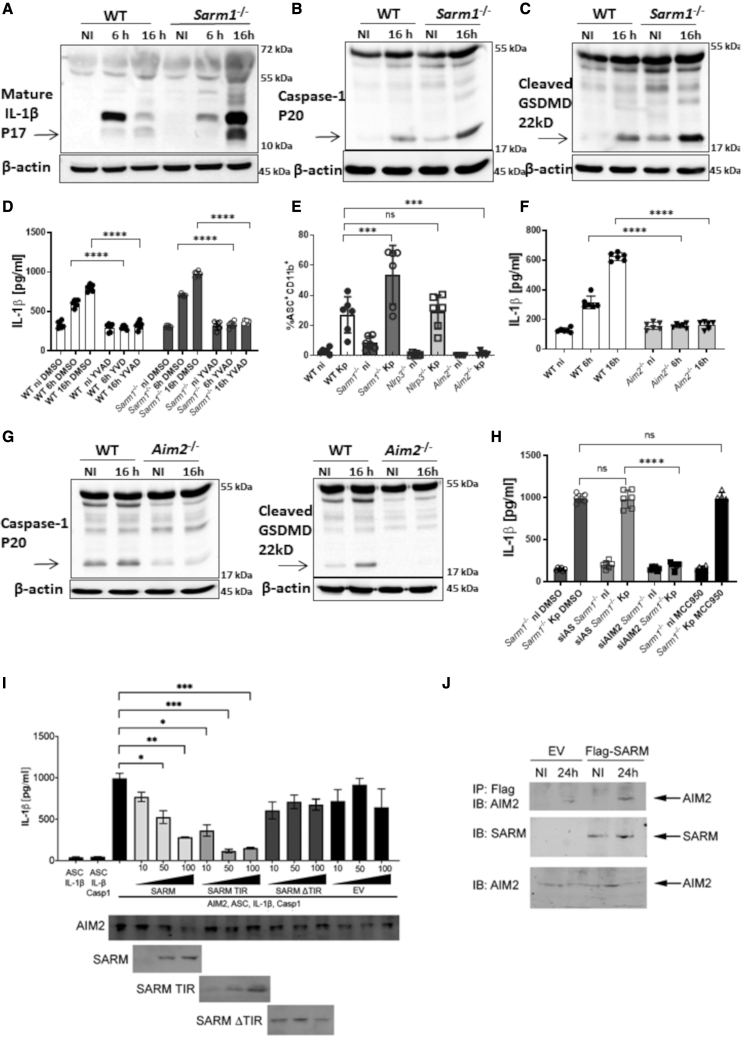
SARM1 negatively regulates *K. pneumoniae*-induced AIM2 inflammasome activation (A–C) Immunoblot analysis of processed pro-IL-1β (A), processed caspase-1 (B), and cleaved gasdermin D (GSDMD) (C) in infected cells. (D) ELISA of IL-1β secreted by infected cells. Cells were treated with the caspase-1 inhibitor YVAD or the DMSO vehicle solution. (E) ASC specks were detected by flow cytometry 16 h post infection in infected cells. (F) ELISA of IL-1β secreted by infected cells. (G) Immunoblot analysis of processed caspase-1, and cleaved gasdermin D (GSDMD) in infected cells for 16 h. (H) ELISA of IL-1β secreted by *sarm1*^*−/−*^ macrophages treated with the NLRP3 inhibitor MCC950 or DMSO vehicle control, and *sarm1*^*−/−*^ cells transfected with All Stars siRNA control (AS), or Aim2 siRNA (siAim2). Cells were non-infected (ni) or infected with Kp52145 (Kp) for 16 h. (I) Reconstitution of AIM2 inflammasome activation in HEK293T cells by co-transfection of plasmids expressing HA-AIM2, ASC, procaspase-1, and pro-IL-1β. Plasmids expressing FLAG SARM1, FLAG SARM1 TIR, FLAG SARM1 ΔTIR (10, 50, 100 ng), or empty vector (EV) were co-transfected. Secreted IL-1β in the culture supernatants was detected by ELISA. HA-AIM2 and FLAG SARM1 (or truncations) were detected by immunoblotting with anti-HA and anti-FLAG antibodies respectively. (J) *Sarm1*^*−/−*^ iBMDMs expressing empty vector (EV) or FLAG-SARM1 were non-infected (NI) or infected with Kp52145 for 24 h. Immunoprecipitation was performed using anti-FLAG (M2) beads. The immune complexes were detected by immunoblotting with anti-SARM1, anti-AIM2 antibodies. In (A)–(C), (G), and (J) images are representative of three independent experiments. In (D)–(F), (H), and (I) values are presented as the mean ± SD of three independent experiments measured in duplicate. ^∗∗∗∗^p ≤ 0.0001, ^∗∗∗^p ≤ 0.001, ^∗∗^p ≤ 0.01, ^∗^p ≤ 0.05; ns, p > 0.05 for the indicated comparisons determined using one way-ANOVA with Bonferroni contrast for multiple comparisons test.

The increased production of IL-1β by KP-infected *sarm1*^*−/−*^ macrophages led us to characterize the effect of SARM1 on inflammasome activation. Immunoblotting experiments showed elevated levels of cleavage of pro-IL-1β (Figure 3A), and an increased activation of caspase-1 in infected *sarm1*^*−/−*^ macrophages compared with infected wild-type cells (Figure 3B). Absence of SARM1 resulted in enhanced levels of processed GSDMD upon infection (Figure 3C). The use of the caspase-1 inhibitor YVAD (Motani et al., 2011) confirmed that the release of IL-1β by KP-infected cells was caspase-1 dependent (Figure 3D). IL-1β release is ASC and GSDMD dependent because we found significantly decreased levels of IL-1β in the supernatants of infected *asc*^*−/−*^ and *gsdmd*^*−/−*^ macrophages compared with infected wild-type cells (Figure S5A). Immunoblotting experiments showed a decrease in the levels of processed pro-IL-1β in the supernatants of infected *asc*^*−/−*^ and *gsdmd*^*−/−*^ macrophages (Figure S5B). Together, these results are consistent with enhanced inflammasome activation in KP-infected *sarm1*^*−/−*^ macrophages. We next examined whether absence of SARM1 affects ASC speck formation. After inflammasome activation, ASC oligomerizes in large protein aggregates enabling the subsequent clustering of caspase-1 (Cai et al., 2014; Lu et al., 2014). Therefore, detection of ASC specks is a distinguishing feature of inflammasome activation. Single-cell analysis by flow cytometry revealed that a greater percentage of cells displayed ASC-speck formation in Kp52145-infected *sarm1*^*−/−*^ macrophages (Figure 3E). Collectively, these results show that SARM1 negatively regulates KP-induced inflammasome activation.

Inflammasome-mediated activation of caspase-1 also leads to pyroptosis. We assessed pyroptosis by the uptake of the vital dye neutral red (Repetto et al., 2008). This cationic dye penetrates cell membranes by non-ionic passive diffusion and concentrates in the lysosomes. The uptake of neutral red depends on the cell’s capacity to maintain pH gradients through the production of ATP. Kp52145 triggered cell death in wild-type macrophages (Figure S6). The infection of *casp-1*^−/−^ and *gsdmd*^−/−^ cells confirmed that Kp52145-induced cell death was caspase-1 and GSDMD dependent (Figure S6A). However, we observed no cell death in infected *sarm1*^*−/−*^ cells (Figure S6B) despite the increased activation of caspase-1. This finding is in agreement with the data showing that SARM1 is required for pyroptosis upon inflammasome activation following LPS priming (Carty et al., 2019). Altogether, these data suggest that, following KP infection, IL-1β secretion and pyroptosis are uncoupled in the absence of SARM1.

We next sought to identify the inflammasome regulated by SARM1 in Kp52145-infected macrophages. SARM1 inhibits NLRP3 (Carty et al., 2019); therefore, we asked whether NLRP3 mediates the secretion of IL-1β in KP-infected macrophages. However, the NLRP3 inhibitor MCC950 (Coll et al., 2015) did not reduce Kp52145-induced secretion of IL-1β (Figure S5C). We found no reduction in IL-1β levels in the supernatants of infected *nlrp3*^*−/−*^ macrophages compared with wild-type cells (Figure S5D), and no decrease in the levels of cleavage pro-IL-1β (Figure S5E). Kp52145 infection even increased the levels of NLRP3 (Figure S5F). Altogether, these data demonstrate that NLRP3 is not required for KP induction of IL-1β. Although NLRC4 mediates IL-1β secretion following infection with other Gram-negative pathogens, we consider it unlikely that KP activates NLRC4 because KP does not express any of the bacterial proteins known to activate this inflammasome. We next considered whether AIM2, also activated by Gram-negative pathogens (Ge et al., 2012; Rathinam et al., 2010; Tsuchiya et al., 2010), mediates KP*-*induced release of IL-1β. Indeed, IL-1β release was abrogated in Kp52145-infected *aim2*^*−/−*^ macrophages (Figure 3F). Neither caspase-1 nor GSDMD were processed in infected *aim2*^*−/−*^ macrophages (Figure 3G). ASC-speck formation was not detected in infected *aim2*^*−/−*^ cells in contrast to infected wild type and *nlrp3*^*−/−*^ cells (Figure 3E). The percentage of cells with ASC-specks was not significantly different between wild type and *nlrp3*^*−/−*^ macrophages, corroborating further that KP does not activate the NLRP3 inflammasome (Figure 3E). As anticipated, Kp52145 induced pyroptosis in *nlrp3*^*−/−*^ cells, whereas this was not the case in infected *aim2*^*−/−*^ macrophages (Figure S6A). Collectively, this evidence demonstrates that AIM2 is the inflammasome mediating IL-1β release and pyroptosis following KP infection. However, the possibility exists that other inflammasome(s) might be activated in the absence of SARM1. To confirm that indeed AIM2 mediates IL-1β secretion in Kp52145-infected *sarm1*^*−/−*^ macrophages, we reduced *aim2* levels by siRNA in *sarm1*^*−/−*^ macrophages. Control experiments confirmed the knockdown efficiency (Figure S5G). We found a reduction in IL-1β levels in the supernatants of *aim2* knockdown cells compared with All Stars siRNA transfected control cells (Figure 3H). Treatment of infected *sarm1*^*−/−*^ macrophages with the NLRP3 inhibitor MCC950 did not result in any decrease in IL-1β levels (Figure 3H), indicating that KP does not activate NLRP3 even in the absence of SARM1.

To examine whether SARM1 had a direct effect on AIM2, we reconstituted the AIM2 inflammasome in HEK293 cells by transfecting plasmids expressing pro-IL-1β, pro-caspase-1, ASC, and AIM2. Under these conditions, the inflammasome induces the secretion of IL-1β without external stimulus (Shi et al., 2016), and this is AIM2 dependent since no detectable mature IL-1β was produced from cells transfected with all the inflammasome components except AIM2 (Figure 3I). AIM2-dependent secretion of IL-1β was inhibited by the expression of SARM1 (Figure 3I). We next determined which domains of SARM1 were required for AIM2 inhibition by expressing different truncations of SARM1. Results showed that the TIR domain alone was sufficient to inhibit IL-1β release (Figure 3I). These data led us to determine whether KP induces the interaction between SARM1 and AIM2 to inhibit inflammasome activation. Co-immunoprecipitation experiments infecting retrovirally transfected FLAG-SARM1 in *sarm1*^*−/−*^ macrophages showed that SARM1 immunoprecipitated AIM2 only in Kp52145-infected cells (Figure 3J).

Altogether, we propose that KP exploits SARM1 to inhibit AIM2 inflammasome activation by a direct interaction between SARM1 and AIM2.

### KP induces AIM2 in a type I IFN-dependent manner

**Figure 4:**
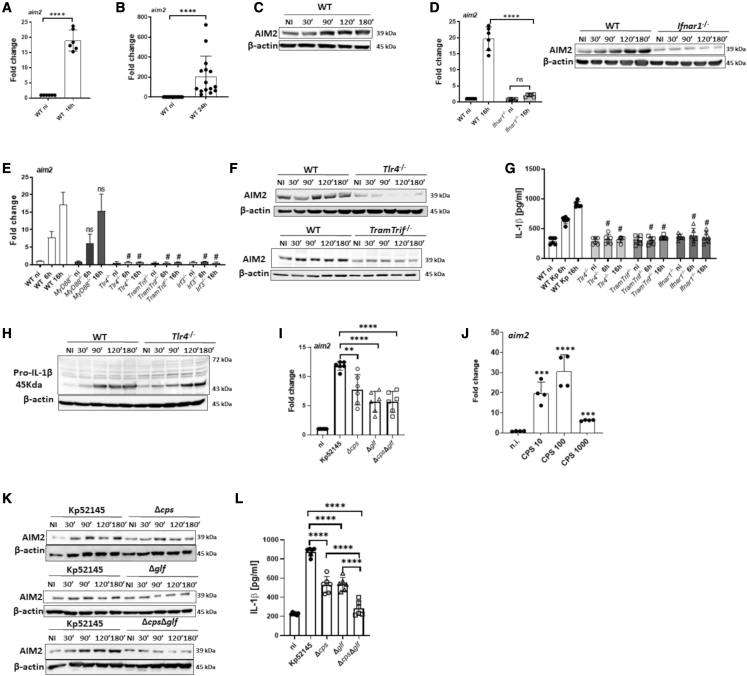
*K. pneumoniae* induces AIM2 in a type I IFN-dependent manner (A) *aim2* mRNA in infected cells for 16 h. (B) *aim2* mRNA levels in the lungs of infected WT mice for 24 h. (C) Immunoblot analysis of AIM2 and β-actin following infection. (D) *aim2* mRNA in infected cells for 16 h. Immunoblot analysis of AIM2 following infection. (E) *aim2* mRNA levels in infected cells for 6 and 16 h. (F) Immunoblot analysis of AIM2 and β-actin levels in infected cells. (G) ELISA of IL-1β secreted by infected cells. (H) Immunoblot analysis of pro-IL-1β and β-actin levels in infected cells. (I) *aim2* mRNA levels in cells infected with Kp52145, the capsule mutant 52145-Δ*manC* (Δ*cps*), the mutant lacking the LPS O-polysaccharide, 52145-Δ*glf* (Δ*glf*), and the double mutant lacking the CPS and the LPS O-polysaccharide, 52145-Δ*wca*_*k2*_– Δ*glf* (Δ*cps* Δ*glf*) for 16 h. (J) *aim2* mRNA levels in WT cells challenged with increasing concentrations purified *K. pneumoniae* capsule (CPS 10, 10 ng/mL; CPS 100, 100 ng/mL, and CPS 1000, 1,000 ng/mL) for 24 h. (K) Immunoblot analysis of AIM2 and β-actin levels in cells infected with Kp52145, the capsule mutant 52145-Δ*manC* (Δ*cps*), the mutant lacking the LPS O-polysaccharide, 52145-Δ*glf* (Δ*glf*), and the double mutant lacking the CPS and the LPS O-polysaccharide, 52145-Δ*wca*_*k2*_– Δ*glf* (Δ*cps* Δ*glf*). (L) ELISA of IL-1β secreted by cells infected with Kp52145 the capsule mutant 52145-Δ*manC* (Δ*cps*), the mutant lacking the LPS O-polysaccharide, 52145-Δ*glf* (Δ*glf*), and the double mutant lacking the CPS and the LPS O-polysaccharide, 52145-Δ*wca*_*k2*_*–* Δ*glf* (Δ*cps* Δ*glf*) 16 h. In (A), (B), (D), (E), (G), and (I)–(L) values are presented as the mean ± SD of three independent experiments measured in duplicate. ^∗∗∗∗^p ≤ 0.0001; ns, p > 0.05 for the indicated comparisons determined using unpaired t test (A and B) or one-way ANOVA with Bonferroni contrast for multiple comparisons test (D). In (E) and (G), #p ≤ 0.0001; ns, p > 0.05 for the comparison between knockout and WT cells at 6 or 16 h post infection using one way-ANOVA with Bonferroni contrast for multiple comparisons test.

We next sought to investigate whether KP infection affects the expression levels of AIM2. Kp52145 induced the expression of *aim2 in vitro* (Figure 4A) and in the lungs of infected mice (Figure 4B). Western blot experiments demonstrated that Kp52145 increased the expression of AIM2 in wild-type macrophages (Figure 4C). We next investigated the signaling pathways governing KP induction of *aim2*. *Aim2* has been identified as an ISG (Fernandes-Alnemri et al., 2009), and the interferome prediction tool (Rusinova et al., 2013) indicates that type I IFN activates the expression of *aim2* in human and mouse cells. Consistent with this prediction, *aim2* and AIM2 levels were reduced in KP-infected *ifnar1*^*−/−*^ cells (Figure 4D). We then tested whether KP induces *aim2* and AIM2 in cells deficient for the TLR4-TRAM-TRIF-IRF3 pathway mediating type I IFN production induced by KP (Ivin et al., 2017). Kp52145 did not increase *aim2* levels in *tlr4*^*−/−*^, *tram*^*−/−*^*trif*^*−/−*^, and *irf3*^*−/−*^ macrophages (Figure 4E). No significant differences were found between infected wild-type and *myd88*^*−/−*^ macrophages (Figure 4E). Kp52145 did not increase AIM2 levels in *tlr4*^*−/−*^ and *tram*^*−/−*^*trif*^*−/−*^ macrophages (Figure 4F). We next determined IL-1β production in cells deficient for the signaling pathway mediating type I IFN production in KP-infected macrophages. Kp52145 did not induce the release of IL-1β in *tlr4*^*−/−*^, *tram*^*−/−*^*trif*^*−/−*^, and *ifnar1*^*−/−*^ macrophages (Figure 4G). Pro-ILβ production was not significantly reduced in infected *tlr4*^*−/−*^ cells, ruling out that the lack of IL-1β production in the absence of TLR4 was due to reduced levels of pro-IL-1β (Figure 4H).

These results led us to investigate whether the capsule polysaccharide (CPS), and LPS O-polysaccharide, mediating the production of type I IFN following KP infection (Ivin et al., 2017), are involved in *aim2* induction. Cells were infected with single mutants lacking each of the polysaccharides, and a double mutant lacking both (Ivin et al., 2017; Sa-Pessoa et al., 2020). The three mutants induced less *aim2* than the wild-type strain (Figure 4I). Purified CPS induced *aim2* (Figure 4J). Immunoblotting analysis showed that the three mutants did not increase AIM2 levels (Figure 4K). As anticipated, the CPS and LPS O-polysaccharide mutants induced less IL-1β than the wild-type strain, the double mutant being the strain inducing the lowest IL-1β levels (Figure 4L).

Collectively, these results demonstrate that signaling via IFNAR1 is required for AIM2 activation by KP upon recognition of the CPS and the LPS O-polysaccharide by TLR4.

### KP induces SARM1 in a type I IFN-dependent manner

**Figure 5:**
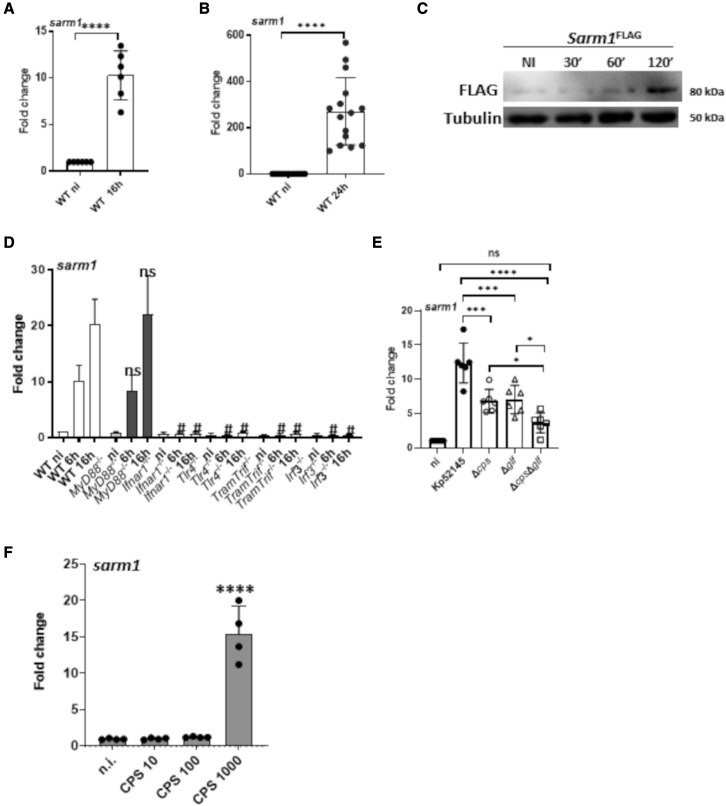
*K. pneumoniae* induces SARM1 in a type I IFN-dependent manner (A) *sarm1* mRNA levels in infected cells for 16 h. (B) *sarm1* mRNA levels in the lungs of infected WT mice for 24 h. (C) Immunoblot analysis of SARM1-FLAG in infected cells. (D) *sarm1* mRNA levels in infected cells for 6 and 16 h. (E) *sarm1* mRNA levels in infected WT cells with Kp52145, the capsule mutant 52145-Δ*manC* (Δ*cps*), the mutant lacking the LPS O-polysaccharide, 52145-Δ*glf* (Δ*glf*), the double mutant lacking the CPS, and the LPS O-polysaccharide, 52145-Δ*wca*_*k2*_– Δ*glf* (Δ*cps* Δ*glf*) for 16 h. (F) *sarm1* mRNA levels in WT cells challenged with increasing concentrations purified *K. pneumoniae* capsule (CPS 10, 10 ng/mL; CPS 100, 100 ng/mL, and CPS 1000, 1,000 ng/mL) for 24 h. In (C) the image is representative of three independent experiments. In (A), (B), and (D)–(F) the values are presented as the mean ± SD of three independent experiments measured in duplicate. In (A), (B), and (D) ^∗∗∗∗^p ≤ 0.0001, ^∗∗∗^p ≤ 0.001, ^∗^p ≤ 0.05 for the indicated comparisons; in (C) #p ≤ 0.0001; ns, p > 0.05 for the comparisons between the knockout and WT cells at the same time point post infection. Significance was established using one-way ANOVA with Bonferroni contrast for multiple comparisons test.

It is common for pathogens to upregulate or activate the expression of the host proteins they target for their own benefit. It might be then expected that KP upregulates the expression of SARM1. Indeed, Kp52145 induced the expression of *sarm1 in vitro* (Figure 5A) and in the lungs of infected mice (Figure 5B). Infection of *Sarm1*^*FLAG*^ cells confirmed that Kp52145 increased the expression of SARM1 (Figure 5C). We next sought to identify the signaling pathways governing KP induction of *sarm1.* Analysis of the promoter region of SARM1 interrogating the interferome database (Rusinova et al., 2013) identified SARM1 as an ISG. We then speculated that KP regulates SARM1 in a type I IFN-dependent manner. Providing initial support to this notion, Kp52145 did not induce *sarm1* in *ifnar1*^*−/−*^ macrophages (Figure 5D). Furthermore, *sarm1* levels were reduced in infected *tlr4*^*−/−*^, *tram*^*−/−*^*trif*^*−/−*^, and *irf3*^*−/−*^ macrophages (Figure 5D). In contrast, Kp52145 induced *sarm1* in *myd88*^*−/−*^ macrophages (Figure 5D). These results led us to investigate whether the CPS and the LPS O-polysaccharide are involved in *sarm1* induction. The three mutants induced less *sarm1* than the wild-type strain, although the double mutant lacking CPS and the LPS O-polysaccharide induced less *sarm1* than each of the single mutants (Figure 5E). The levels of *sarm1* induced by the double mutant were not significantly different than those of non-infected cells (Figure 5E). Purified CPS increased the levels of *sarm1* (Figure 5F).

Altogether, these results confirm experimentally that KP leverages type I IFN signaling to induce SARM1 following activation of the TLR4-TRAM-TRIF-IRF3 pathway upon recognition of the CPS and the LPS O-polysaccharide.

### SARM1 promotes KP virulence

**Figure 6:**
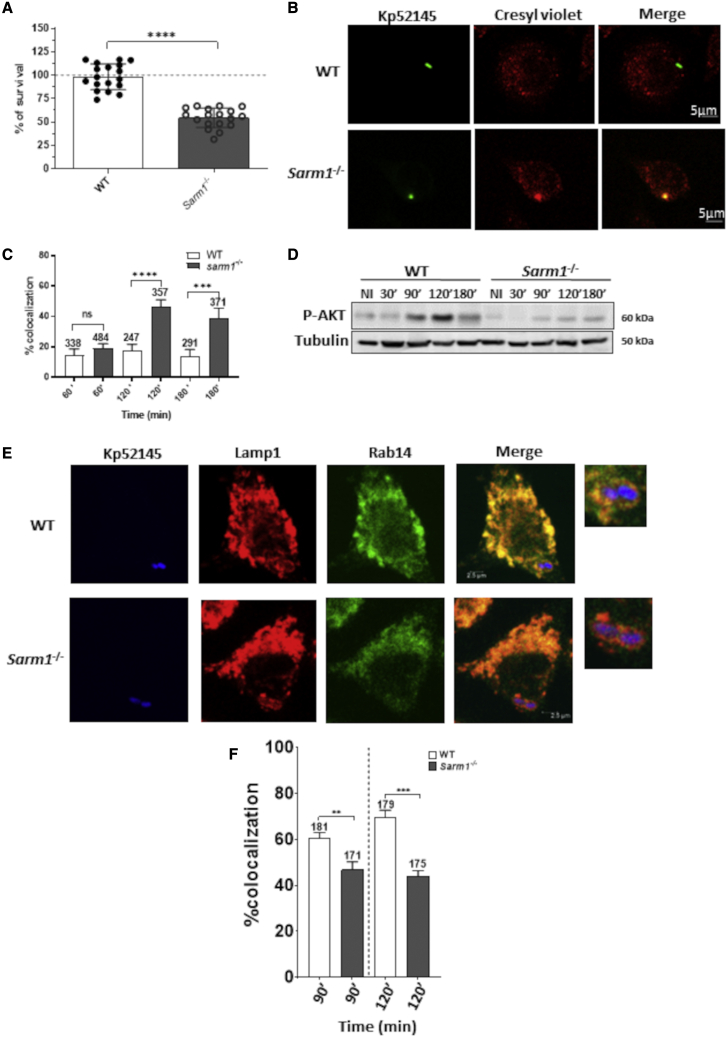
SARM1 is required for *K. pneumoniae* intracellular survival (A) Kp52145 intracellular survival in WT (WT) and *sarm1*^*−/−*^ 4 h after addition of gentamycin (30 min of contact). Results are expressed as percent of survival (CFUs at 4 h versus 1 h in *sarm1*^−/−^ cells normalized to the results obtained in WT macrophages set to 100%). Values are presented as the mean ± SD of six independent experiments measured in triplicate. (B) Immunofluorescence confocal microscopy of the colocalization of Kp52145 harboring pFPV25.1Cm and cresyl violet in cells. The images were taken 90 min post infection. Images are representative of duplicate coverslips in three independent experiments. (C) Percentage of Kp52145 harboring pFPV25.1Cm colocalization with cresyl violet over a time course. Values are given as mean percentage of Kp52145 colocalizing with the marker ± SD. The number of infected cells counted per time in three independent experiments are indicated in the figure. (D) Immunoblot analysis of phosphorylated Akt (P-AKT) following infection with Kp52145. (E) Immunofluorescence confocal microscopy of the colocalization of Kp52145 harboring pFPV25.1Cm, Lamp1, and Rab14 in WT and *sarm1*^*−/−*^ macrophages. The images were taken 90 min post infection. Images are representative of duplicate coverslips in three independent experiments. (F) Percentage of Kp52145 harboring pFPV25.1Cm colocalization with Lamp1 and Rab14 over a time course. Values are given as mean percentage of Kp52145 colocalizing with the marker ± SD. The number of infected cells counted per time in three independent experiments are indicated in the figure. In (A, C and F) the values are presented as the mean ± SD of three independent experiments measured in duplicate. ^∗∗∗∗^p ≤ 0.0001, ^∗∗∗^p ≤ 0.001, ^∗∗^p ≤ 0.01; ns, p > 0.05 for the indicated comparisons determined using unpaired t test.

We next sought to investigate whether SARM1 contributes to KP subversion of cell-autonomous immunity. KP manipulates the phagosome traffic following phagocytosis to create a unique niche that does not fuse with lysosomes, the KCV, allowing the intracellular survival of KP (Cano et al., 2015). We asked whether the absence of SARM1 impairs KP intracellular survival. The attachment of Kp52145 was not affected in *sarm1*^*−/−*^ cells (Figure S7A), whereas there was a slight reduction in the number of engulfed bacteria (Figure S7B). Time-course experiments revealed that the intracellular survival of Kp52145 was significantly reduced in *sarm1*^*−/−*^ macrophages (Figure 6A). We then sought to determine whether the reduced intracellular survival was due to an increase in the colocalization of lysosomes with the KCV. Lysosomes were labeled with the membrane-permeant fluorophore cresyl violet (Ostrowski et al., 2016), and cells were infected with GFP-labelled Kp52145 to assess the KCV at the single-cell level by immunofluorescence. Confocal microscopy experiments revealed that the majority of the KCVs from wild-type macrophages did not colocalize with cresyl violet (Figures 6B and 6C), corroborating our previous work (Cano et al., 2015). In contrast, there was an increase in the colocalization of the KCVs from *sarm1*^*−/−*^ macrophages with cresyl violet (Figures 6B and 6C), demonstrating that the absence of SARM1 results in the fusion of the KCV with lysosomes with a concomitant reduction in the numbers of intracellular bacteria.

KP targets the PI3K-AKT axis to survive intracellularly (Cano et al., 2015). We asked whether the absence of SARM1 would affect KP-induced AKT phosphorylation. Immunoblotting experiments confirmed that Kp52145-induced AKT phosphorylation was reduced in *sarm1*^*−/−*^ macrophages compared with wild-type cells (Figure 6D). In KP-infected cells, AKT activation is linked to the recruitment of Rab14 to the KCV to block the fusion with lysosomes (Cano et al., 2015). We then investigated the recruitment of Rab14 to the KCV in *sarm1*^*−/−*^ macrophages. Figures 6E and 6F illustrate that Rab14 does not colocalize with the KCV in *sarm1*^*−/−*^ macrophages in contrast to wild-type macrophages. Altogether, this evidence demonstrates that SARM1 is crucial for KP-induced activation of the PI3K-AKT-Rab14 axis to control the phagosome maturation to survive inside macrophages.

**Figure 7:**
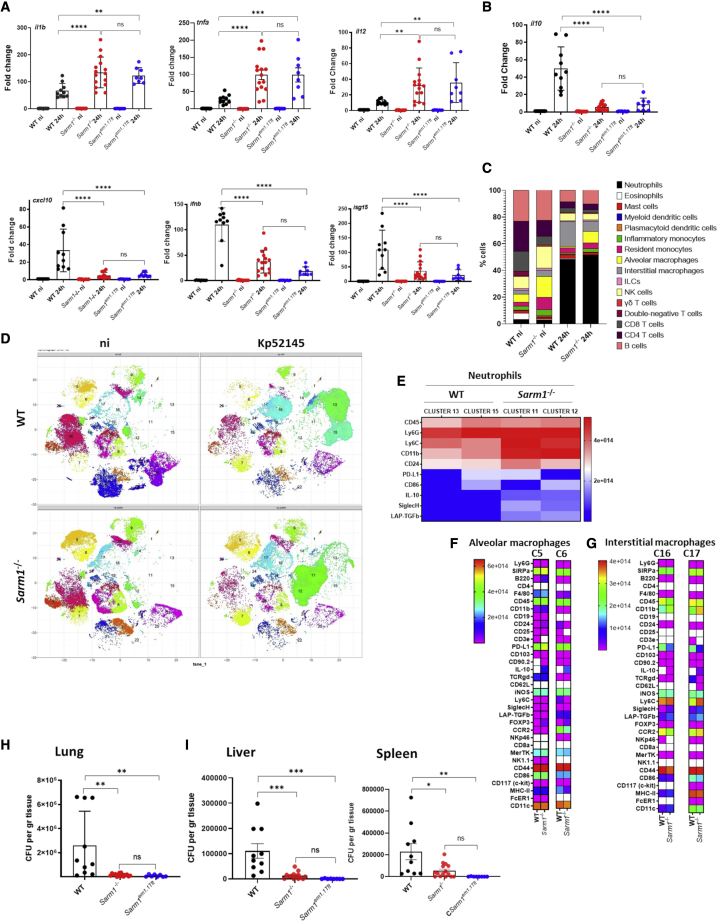
SARM1 promotes *K. pneumoniae* virulence (A and B) *il1b*, *tnfa*, *il12*, *cxcl10*, *ifnb*, and *isg15* mRNA (A) and *il10* (B) in the lungs of infected mice for 24 h. Each dot represents a different mouse. (C) Percentage of immune cells in the lungs of WT and *sarm1*^*−/−*^ mice non-infected (ni) or infected for 24 h. Results are based on data from three mice per group. (D) PhenoGraph cluster analysis of immune populations in the lungs WT, and *sarm1*^*−/−*^ mice non-infected (ni) or infected for 24 h. Results are based on data from three mice per group. (E) Heatmap showing relative signal intensities of the indicated markers on neutrophils of clusters 13 and 15 found in the lungs of infected WT mice, and clusters 11 and 13 detected in the lungs of *sarm1*^*−/−*^ mice. The heatmap is colored based on signal intensity of the indicated markers. Results are based on data from three mice per group. (F) Heatmap showing relative signal intensities of the indicated markers on alveolar macrophages of clusters 5 and 6 found in the lungs of infected WT and *sarm1*^*−/−*^ mice. The heatmap is colored based on signal intensity of the indicated markers. Results are based on data from three mice per group. (G) Heatmap showing relative signal intensities of the indicated markers on interstitial macrophages of clusters 16 and 17 found in the lungs of infected WT and *sarm1*^*−/−*^ mice. The heatmap is colored based on signal intensity of the indicated markers. Results are based on data from three mice per group. (H) Bacterial load in the lungs of infected WT mice, *sarm1*^*−/−*^, and *Sarm1*^*em1.1Tft*^ for 24 h. Each dot represents a different mouse. (I) Bacterial load in the livers and spleens of infected WT mice, *sarm1*^*−/−*^, and *Sarm1*^*em1.1Tft*^ for 24 h. Each dot represents a different mouse. In (A), (B), (H), and (I) the values are presented as the mean ± SD of three independent experiments measured in duplicate. ^∗∗∗∗^p ≤ 0.0001, ^∗∗∗^p ≤ 0.001, ^∗∗^p ≤ 0.01, ^∗^p ≤ 0.05; ns, p > 0.05 for the indicated comparisons using one-way ANOVA with Bonferroni contrast for multiple comparisons test.

To obtain a global view of the role of SARM1 in KP infection biology, we examined the contribution of SARM1 to modulate the inflammatory responses induced by KP *in vivo*. Kp52145 induced the expression of *il1b*, *tnfa*, *il12*, *cxcl10 ifnb*, and *isg15 in vivo* (Figure 7A), although the levels of *il1b*, *tnfa*, and *il12* were higher in *sarm1*^*−/−*^ mice than in wild-type ones (Figure 7A). We observed a significant decrease in the levels of *il10* in *sarm1*^*−/−*^ mice compared with wild-type ones (Figure 7B). Similar results were obtained infecting *Sarm1*^*em1.1Tft*^ mice, indicating that the results are neither dependent on the mouse strain nor on the way the *sarm1* knockout mice were generated (Figures 7A and 7B). Together, these results demonstrate that the absence of SARM1 results in heightened inflammation following KP infection *in vivo*.

We used mass cytometry to profile the immune cells of infected and non-infected mice. We tested a panel of 33 surface and intracellular markers that would enable resolution of 100 lymphoid and myeloid cell types (Table S1). The clustering algorithm PhenoGraph was used to define cell communities (Levine et al., 2015); 16 populations were identified (Figure 7C). In *sarm1*^*−/−*^ non-infected mice, we found a significant increase in the percentage of resident monocytes (MHC-II-Ly6G-Ly6C^+^CD11b^+^CD11c-CCR2^high^) (p < 0.05) compared with non-infected wild-type mice, whereas there were no significant differences in the percentages of any other immune cell (Figure 7C). Following infection, we observed a significant increase in the percentage of neutrophils (MHC-II^−^Ly6G^+^Ly6C^+^F4/80^−/low^) in both genotypes, although the numbers were not significantly different between them (Figure 7C). In infected wild-type mice, there was an increase in the percentage of interstitial macrophages (MHC-II^+^Ly6G^−^Ly6C^+^CD11b^+^) compared with infected *sarm1*^*−/−*^ mice (p < 0.01) (Figure 7C). In contrast, there was a significant increase in the percentage of alveolar macrophages (MHC-II^+^Ly6G^−^Ly6C^−^CD11b^low^CD11c^+^) (p < 0.01) in infected *sarm1*^*−/−*^ mice compared with infected wild-type ones (Figure 7C). The percentages of alveolar macrophages were not significantly different between infected and non-infected *sarm1*^*−/−*^ mice. There were no significant differences in the percentages of other immune cells between infected genotypes (Figure 7C). Altogether, mass cytometry analysis demonstrated an increase in the numbers of neutrophils and alveolar macrophages in *sarm1*^*−/−*^-infected mice. These cells are crucial in host defense against KP (Broug-Holub et al., 1997; Xiong et al., 2015, 2016; Ye et al., 2001).

For each of these 16 immune cell populations, we performed further cell-type subclustering; in these analyses we only detected substructure among CD4 T cells, CD8 T cells, NK cells, B cells, neutrophils, alveolar macrophages, and interstitial macrophages (Figures 7D and S8A). The heatmap of the markers expressed by each of the clusters is shown in Figure S8B. No differences were found between infected wild-type and *sarm1*^*−/−*^ mice in the CD4 T cells (clusters 3, 21, 23), CD8 T cells (clusters 27 and 25), NK cells (clusters 8, 31, and 28), and B cells (clusters 7, 30, 10, and 2) (Figure 7C; Table S2). In contrast, we observed differences in the subsets of neutrophils, alveolar macrophages, and interstitial macrophages (Figure 7C; Table S2). Within neutrophils, clusters 11 and 12 were only present in *sarm1*^*−/−*^-infected mice, whereas clusters 13 and 15 were only present in wild-type-infected mice. Heatmap analysis of these clusters revealed that each of the clusters can be differentiated based on the expression levels of PD-L1 and CD86 in both genetic backgrounds (Figure 7E). Clusters 11 and 12, only found in *sarm1*^*−/−*^-infected mice, were characterized by the expression levels of the markers Ly6C, CD11b, CD24, IL-10, Siglec H, and LAP-TGFβ (Figure 7E), revealing an increase activation of neutrophils in *sarm1*^*−/−*^ mice following infection. In the case of alveolar macrophages, clusters 5 and 6 (Figures 7C and S8A) were predominant in *sarm1*^−/−^ mice, and they can be differentiated by the expression of CCR2. The expression of CCR2 is higher in cluster 6 than in cluster 5. No major differences were noted between genetic backgrounds with infection, except that *sarm1*^*−/−*^ cells showed an increase in the levels of IL-10 (p < 0.0001) (Figure 7F). Within the population of interstitial macrophages, cluster 16 was the predominant in wild-type-infected mice, whereas cluster 17 was the predominant one in *sarm1*^*−/−*^-infected mice (Figure 7D). The levels of CD11c differentiates both clusters, higher in cluster 17 than in cluster 16 (Figure 7G). High levels of CD11c are associated with the activation of immune cells (Arnold et al., 2016; Lewis et al., 2015). Cells within cluster 17, found in *sarm1*^−/−^ mice, were characterized by high levels of CD11b, iNOS, Ly6C, CCR2, Siglec H, SIRPα, LAP-TGFβ, CD44, CD86. MHC-II, and Cd11c (Figure 7G), all markers of activation of immune cells. In conclusion, the analysis of the subsets of immune populations revealed the presence of different subsets of neutrophils and interstitial macrophages in *sarm1*^*−/−*^-infected mice characterized by elevated levels of markers associated with the activation of immune cells.

Finally, we determined the ability of *sarm1*^*−/−*^ mice to control bacterial growth following intranasal infection. At 24 h post infection, there was a 94% reduction in bacterial load in the lungs of infected *sarm1*^*−/−*^ mice compared with wild-type-infected ones (Figure 7H). We found a lower dissemination of Kp52145 to liver and spleen in *sarm1*^*−/−*^ mice than in wild-type ones (Figure 7I). The infection of *Sarm1*^*em1.1Tft*^ mice yielded similar results; the knockout mice controlled the lung infection more efficiently than the wild-type ones and there was less dissemination to deeper tissues (Figures 7H and 7I). Altogether, this evidence establishes the crucial role of SARM1 for KP survival *in vivo*.

## Discussion

Here, we show KP exploits SARM1 to control MyD88- and TRIF-governed inflammation, to limit the activation of the MAP kinases ERK and JNK, and to induce IL-10 by fine-tuning the p38-type I IFN axis. SARM1 inhibits the activation of the KP-induced AIM2 inflammasome with a concomitant reduction in IL-1β (Figure S9) to suppress further inflammation. SARM1 is necessary for KP intracellular survival whereas, *in vivo*, absence of SARM1 facilitates the clearance of the pathogen. Collectively, our findings reveal one of the Achilles heels of our immune system exploited by KP to overcome host protective responses.

Manipulation of SARM1 is a previously unknown anti-immunology strategy deployed by a human pathogen. This finding is particularly relevant in the case of KP, which does not encode any type III or IV secretion system or any of the toxins implicated in counteracting innate immunity, making then interesting to uncover KP anti-immunology strategies. Previous work from the laboratory demonstrate that KP exploits other proteins regulating cell intrinsic immunity, such as the deubiquitinase CYLD, the MAKP phosphatase MKP-1, and the Sentrin/SUMO-specific protease (SENP) 2, to control cell responses (Regueiro et al., 2011; Sa-Pessoa et al., 2020). The theme taking shape is that a signature of KP infection biology is to hijack proteins controlling immune homeostasis. This strategy is radically different to those exploited by other pathogens, such as *Listeria*, *Salmonella*, *E. coli*, *Brucella*, *Legionella*, and *Shigella*, which deploy bacterial effector proteins to block the activation of cell intrinsic immunity pathways.

This study suggests that KP leverages the TIR-TIR interactions between SARM1 and MyD88 and TRIF, to attenuate MyD88- and TRIF-dependent responses. There are few examples of pathogens exploiting TIR-TIR interactions to blunt the activation of TLR-controlled signaling pathways (Askarian et al., 2014; Cirl et al., 2008; Coronas-Serna et al., 2020; Imbert et al., 2017; Xiong et al., 2019). Without exception, these pathogens deliver a prokaryotic protein containing the TIR domain into immune cells, whereas KP is the first pathogen hijacking an endogenous mammalian TIR-containing protein.

Another novel finding of our work is that the absence of SARM1 impairs KP induction of IL-10. The fact that neutralization of IL-10 enhances the clearance of the pathogen (Greenberger et al., 1995) illustrates the role of the cytokine in KP infection biology. How KP induces IL-10 was unknown. Our data implicate p38 whose activation is fine-tuned by KP-induced type I IFN. The absence of SARM1 increases the levels of type I IFN, reducing the activation of p38 with a concomitant decrease in IL-10. The connection between type I IFNs and IL-10 has been described; however, and in contrast to our results, the data indicate that type I IFN signaling is needed to sustain IL-10 production in macrophages following challenge with LPS or *Mycobacterium* spp. (Chang et al., 2007; McNab et al., 2014; Pattison et al., 2012). These results reflect the importance of type I IFNs levels in the host-pathogen interface, although the consequences are context dependent.

Previous work revealed the importance of IL-1β-governed responses in host defense against KP (Cai et al., 2012). Not surprisingly, KP has evolved to blunt IL-1β-mediated inflammation (Frank et al., 2013; Regueiro et al., 2011). There was no evidence indicating whether KP counteracts inflammasome activation to limit the production of IL-1β. Here, we demonstrate that this is the case. The facts that SARM1 inhibits NLRP3 activation (Carty et al., 2019), and that KP may activate NLRP3 inflammasome (Hua et al., 2015; Willingham et al., 2009), made plausible that SARM1 would inhibit NLRP3 activation in KP-infected cells. However, this was not the case even in infected *sarm1*^*−/−*^ cells. This is even more puzzling considering that KP increased NLRP3 expression and that the stimuli reported to activate NLRP3, such as ROS, are most likely present in KP-infected cells. It is then tempting to speculate that KP has evolved mechanisms to blunt the activation of NLRP3.

Our data demonstrate that AIM2 is the inflammasome inhibited by SARM1 in KP-infected cells. Components of the type I IFN signaling pathway were essential for the activation of AIM2 inflammasome by KP. This is similar to *Listeria monocytogenes* and *Francisella* spp, two other pathogens activating AIM2 (Fernandes-Alnemri et al., 2010; Henry et al., 2007; Jones et al., 2010; Man et al., 2015; Rathinam et al., 2010; Tsuchiya et al., 2010). However, cGAS-STING-IRF3-IFNAR1 signaling is necessary in the case of *Listeria-* and *Francisella*-mediated activation of AIM2 (Fernandes-Alnemri et al., 2010; Hansen et al., 2014; Man et al., 2015; Rathinam et al., 2010), whereas TLR4-TRAM-TRIF-IRF3-IFNAR1 mediates KP induction of AIM2. This evidence uncovers the crucial role of IRF3-IFNAR1 signaling in the host-bacteria interface. Recently, we have demonstrated the importance of this hub to control KP infections (Ivin et al., 2017).

Mechanistically, KP triggered an association between SARM1 and AIM2, and the SARM1 TIR domain was sufficient to inhibit AIM2 activation. Our data are consistent with a model in which SARM1 targets AIM2 to suppress the recruitment of ASC and ASC-speck formation, restraining the activation of caspase-1. We previously showed that SARM1 antagonizes the NLRP3 inflammasome by preventing ASC oligomerization, that SARM1 associates with both NLRP3 and ASC, and that NLRP3 and ASC interact more strongly in the absence of SARM1 (Carty et al., 2019). There, the SARM1 TIR domain was found to be essential, and sufficient, for NLRP3 inhibition (Carty et al., 2019). Similarly, here we showed that SARM1 interacts with AIM2 (and only in the context of KP infection), and that the TIR domain of SARM1 was required, and sufficient, for inhibition of AIM2 inflammasome activity. Altogether, the data on NLRP3 and AIM2 suggest that since both NLRP3 and AIM2 have PYRIN domains that mediate interaction with ASC, the SARM1 TIR domain likely interacts with and antagonizes the PYRIN domain of AIM2 or NLRP3 to suppress interaction with ASC. Furthermore, in the case of both NLRP3 and AIM2 the data are consistent with SARM1 also or alternatively interacting with the PYRIN domain of ASC to antagonize ASC oligomerization. Altogether, this evidence supports a direct interaction between the TIR and the PYRIN domains. However, structural data are still needed to validate rigorously this novel interaction between both domains. Our results do not rule out the possibility of an indirect interaction between both domains by a yet to be discovered bridging protein. That the TIR domain of SARM1 is required to antagonize inflammasomes is consistent with the fact that an *E. coli* TIR protein, TcpC, was shown to inhibit NLRP3 inflammasome activity via its TIR domain binding to NLRP3 (Waldhuber et al., 2016).

To the best of our knowledge, KP is the first pathogen deploying a strategy to target directly AIM2 activation because the other known examples are based on reducing the activating signal (Ge et al., 2012; Ulland et al., 2010). The strategy deployed by KP is reminiscent of how cells avoid an excessive activation of AIM2 by leveraging two small proteins, p202 in mouse and IFI16β in human cells, that impede AIM2-ASC complex formation (Wang et al., 2018; Yin et al., 2013).

Except in neurons, the levels of SARM1 are low in most cells types (Doran et al., 2021; Uhlen et al., 2010), suggesting that SARM1 levels are under tight control. We demonstrate that KP induced SARM1 in a type I IFN-dependent manner via a TLR4-TRAM-TRIF-IRF3-IFNAR1 signaling pathway, placing SARM1 as an ISG. Similar to SARM1, type I IFNs are also conserved during evolution and appear in the first vertebrates (Secombes and Zou, 2017), suggesting that KP manipulates an ancient SARM1-type I IFNs axis to counteract the activation of host defences. It is interesting to note the complex interface between KP and type I IFN. On the one hand, TRIF-mediated type I IFN is essential for host defense against KP (Cai et al., 2009; Ivin et al., 2017), including the expression of IL-1β as a result of AIM2 activation (this work), and to limit the production of IL-10 (this work). On the other hand, KP exploits type I IFN to induce SARM1 to attenuate TRIF and AIM2 activation. This evidence supports the notion that there is a threshold of type I IFN levels that needs to be reached to exert a protective role, whereas below this threshold type I IFNs promote KP infection. In this scenario, SARM1 is one of the breaks that KP uses to control type I IFNs.

We were keen to identify the bacterial factor(s) mediating the expression of SARM1. Our results establish that the CPS and the LPS O-polysaccharide induced the expression of SARM1. This is in perfect agreement with the evidence demonstrating that both polysaccharides trigger the production of type I IFNs following the activation of the TLR4-TRAM-TRIF-IRF3-IFNAR1 pathway (Ivin et al., 2017). Importantly, these polysaccharides are required for KP survival in mice (pneumonia model) (Cortes et al., 2002; Lawlor et al., 2005; Tomas et al., 2015), underlining the importance of SARM1 induction as a KP virulence trait since this process is abrogated in these mutant strains. We recently demonstrated that both polysaccharides reduce the SUMOylation of proteins to limit host defense responses involving type I IFN-regulated miRNAs of the *let-7* family (Sa-Pessoa et al., 2020). Altogether, this evidence underscores the role of KP CPS and LPS to hijack regulators of the host immune system, adding to their well-established role in KP stealth behavior (Bengoechea and Sa Pessoa, 2019).

Absence of SARM1 impaired KP-induced activation of AKT, which in turn limited the recruitment of Rab14 to the KCV resulting in the fusion of the KCV with lysosomes (Cano et al., 2015 and this work), impairing KP intracellular survival. It is intriguing to note that two other pathogens, *Salmonella typhimurium* and *M. tuberculosis*, also manipulate the PI3K-AKT-Rab14 pathway to arrest phagosome maturation (Kuijl et al., 2007; Kyei et al., 2006). It is tempting to postulate that SARM1 may also play an important role In the intracellular survival of these two pathogens. If this is the case, the axis SARM1-PI3K-AKT-Rab14 will become one of the central nodes targeted by pathogens to take control over cellular functions.

The fact that *sarm1*-deficient mice were more efficient at controlling KP infection than wild-type mice indicates that KP leverages SARM1 to counteract host defences. The *in vivo* data support that KP exploits SARM1 to limit inflammatory cytokines and chemokines, and to produce IL-10, mirroring the *in vitro* results. A wealth of evidence supports that this lung inflammatory environment is essential to clear KP infections (Bengoechea and Sa Pessoa, 2019). It can be then concluded that KP exploits SARM1 to modify the lung microenvironment to flourish. Mass cytometry analysis uncovered the presence of high numbers of alveolar macrophages, and neutrophils in *sarm1*^*−/−*^-deficient mice. This is in good agreement with previous studies showing the importance of these cell types for the clearance of KP infections (Broug-Holub et al., 1997; Xiong et al., 2015, 2016). Our profile analysis revealed subsets of neutrophils and interstitial macrophages only present in *sarm1*^*−/−*^-infected mice. These cells expressed high levels of markers associated with immune activation further reinforcing the notion that the microenvironment in the absence of SARM1 is hostile for KP.

Our findings indicate that SARM1 is a target to boost human defense mechanisms against KP. SARM1 is a druggable protein, and the crystal structure of the TIR domain of SARM1 is solved at 1.8 Å (Horsefield et al., 2019). This high-resolution structural information should facilitate the development of small-molecule inhibitors. Indeed, efforts are underway to develop pharmacological approaches to inhibit SARM1 to treat diseases with pathophysiological neuronal cell death (DiAntonio, 2019; Hughes et al., 2021). Based on our work, we propose that these drugs will show a beneficial effect to treat KP infections alone or as a synergistic add-on to antibiotic treatment.

### Limitations of this study

We have shown that SARM1 negatively regulates the MyD88- and TRIF-induced inflammatory responses by three different KP strains, one hypervirulent and two multidrug resistant. However, the mechanistic studies probed only the hypervirulent strain. Although we believe that it is unlikely that different KP strains exploit different strategies to hijack SARM1, we cannot rigorously rule out this possibility.

The mass cytometry profiling experiments uncovered subpopulations of neutrophils and interstitial macrophages expressing high levels of activation markers only present in infected *sarm1*^*−/−*^ mice. It will be interesting to characterize in detail each of these subpopulations and to establish their contribution to the clearance of KP by *sarm1*^*−/−*^ mice. A comparative analysis of the immune populations found upon KP infection of different knockout strains that differ in their ability to control KP infection should help to define subpopulations of immune cells associated with KP clearance and those not essential.

## STAR★Methods

### Key resources table

**Table:** 

REAGENT or RESOURCE	SOURCE	IDENTIFIER
**Antibodies**		

anti-IL-1β	R&D Systems	Cat# AF-401-NA, RRID:AB_416684
anti-caspase-1	Cell Signaling	Cat# 24232, RRID:AB_2890194
anti-AIM2	Santa Cruz	Cat# sc-515895; RRID:AB_2922903
anti-NLRP3	Cell Signaling	Cat# 15101; RRID:AB_2922902
anti-Gasdermin-D	Cell Signaling	Cat# 93709; RRID:AB_2800210
anti-Viperin	Novus Biologicals	Cat# NBP2-03971; RRID:AB_2922904
anti-ISG15	Cell Signaling	Cat# 2743: AB_485250
anti-phospho-STAT3	Cell Signaling	Cat# 9145; RRID:AB_2799407
anti-IκBα	Cell Signaling	Cat# 4814; RRID:AB_2797687
anti-phospho-IκBα	Cell Signaling	Cat# 9246; RRID:AB_2922905
anti-phospho-AKT1/2/3	Santa Cruz	Cat# sc-33437; RRID:AB_2922906
anti-phospho-IKKα/β	Cell Signaling	Cat# 2697; RRID:AB_2922907
anti-phospho-IRF3	Cell Signaling	Cat# 4947; RRID:AB_823547
anti-phospho-p-TBK-1/NAK	Cell Signaling	Cat# 5483; RRID:AB_2922908
anti-phospho-JNK	Cell Signaling	Cat# 9251S; RRID:AB_331659
anti-phospho-ERK	Cell Signaling	Cat# 9101; RRID:AB_331772
anti-phospho-p38	Cell Signaling	Cat# 4511; RRID:AB_2797648
anti-Flag M2	Sigma-Aldrich	Cat# F3165; RRID:AB_2922909
anti-HA	Santa Cruz	Cat# sc-805; RRID:AB_2922910
anti α-tubulin	Sigma-Aldrich	Cat# T9026; RRID:AB_2922911
anti-β-actin	Santa Cruz	Cat# sc-130065; RRID:AB_2922912
anti-ASC	Santa Cruz	Cat# sc-22514R; RRID:AB_2922913
anti-Lamp1	Santa Cruz	Cat# sc-19992; RRID:AB_2922914
anti-Rab14	Santa Cruz	Cat# sc-271401; RRID:AB_2922915
Ly6G	Fluidigm	Cat# 3141008B; RRID:AB_2814678
SIRPa	BD Biosciences	Cat# 552371; RRID: AB_394371
B220	Fluidigm	Cat# 3144011B; RRID:AB_2811239
CD4	Fluidigm	Cat# 3145002B; RRID:AB_2687832
F4/80	Fluidigm	Cat# 3146008B; RRID:AB_2895117
CD45	Fluidigm	Cat# 3147003C; RRID:AB_2811243
CD11b	Fluidigm	Cat# 3148003C; RRID:AB_2922916
CD19	Fluidigm	Cat# 3149002B; RRID:AB_2814679
CD24	Fluidigm	Cat# 3150009B; RRID:AB_2922917
CD25	Fluidigm	Cat# 151007B; RRID:AB_2687835
CD3e	Fluidigm	Cat# 3152004B; RRID:AB_2687836
PD-L1	Fluidigm	Cat# 3153016B; RRID:AB_2687837
CD103	BioLegend	Cat# 121402; RRID:AB_535945
CD90.2	Fluidigm	Cat# 3156006B; RRID:AB_2801433
IL-10	Fluidigm	Cat# 3158002B; RRID:AB_2922918
TCRgd	Fluidigm	Cat# 3159012B; RRID:AB_2922919
CD62L	Fluidigm	Cat# 3160008C; RRID:AB_2885021
iNOS	Fluidigm	Cat# 3161011B; RRID:AB_2922920
Ly6C	Fluidigmn	Cat# 3162014B; RRID:AB_2922921
SiglecH	BioLegend	Cat# 129602; RRID:AB_1227757
LAP/TGFb	Fluidigm	Cat# 3164014B; RRID:AB_2687842
FOXP3	Fluidigm	Cat# 3165024A; RRID:AB_2687843
CCR2	R&D Systems	Cat# MAB55381R; RRID:AB_2749828
CD335/NKp46	Fluidigm	Cat# 3167008B; RRID:AB_2922922
CD8a	Fluidigm	Cat# 3168003B; RRID:AB_2811241
MerTK	R&D Systems	Cat# AF591; RRID:AB_2098565
CD161/NK1.1	Fluidigm	Cat# 3170002C: RRID:AB_2885023
CD44	Fluidigm	Cat# 3171003C: RRID:AB_2895121
CD86	Fluidigm	Cat# 3172016B; RRID:AB_2922923
CD117/c-kit	Fluidigm	Cat# 3173004B; RRID:AB_2811230
MHC-II	Fluidigm	Cat# 3174003B; RRID:AB_2922924
FcER1	Fluidigm	Cat# 3176006B; RRID:AB_2922925
CD11c	Fluidigm	Cat# 3209005B; RRID:AB_2811244
CD11b	eBioscience	Cat# 17-0112-82; RRID:AB_469343
CD11c	eBioscience	Cat# 48-0114-82; RRID:AB_1548654

**Bacterial and virus strains**		

*Klebsiellla pneumoniae* CIP52.145	Sansonetti laboratory, Institut Pasteur (France)	PMID: 24885329
*Klebsiella pneumoniae* 52145-Δ*manC*	Bengoechea laboratory, Queen’s University Belfast (UK)	PMID: 28202493
*Klebsiella pneumoniae* 52145-Δ*glf*,	Bengoechea laboratory, Queen’s University Belfast (UK)	PMID: 32994335
*Klebsiella pneumoniae* 52145-Δ*wca*_*k2*_- Δ*glf*	Bengoechea laboratory, Queen’s University Belfast (UK)	PMID: 32994335
*Klebsiella pneumoniae* KP35	Alice Prince laboratory, Columbia University Medical Center (USA)	PMID: 27777978
*Klebsiella pneumoniae* NJST258_2	Frank DeLeo laboratory, National Institute of Allergy and Infectious Diseases (USA)	PMID: 24639510

**Biological samples**		

iBMDM wild-type	BEI Resources	Cat# NR-9456
iBMDM *tlr4*^−/−^	BEI Resources	Cat# NR-9458
iBMDM *myd88*^−/−^	BEI Resoruces	Cat# NR-15633
iBMDM *tram*^*−/−*^*trif*^*−/−*^	BEI Resources	Cat# NR-9568
Anti-SARM1	Icosagen	Bowie laboratory
B16-Blue IFN-α/β reporter cells	InvivoGen	Cat# bb-ifnt1

**Chemicals, peptides, and recombinant proteins**		

MCC950	Sigma-Aldrich	Cat# CAS 256373-96-3
YVAD	Sigma-Aldrich	Cat# CAS 256373-96
Mouse IL10	BioLegend	Cat# 575802
p38 inhibitor SB203580	Sigma-Aldrich	Cat# S8307
mouse anti-IFNAR1 receptor antibody	BioXcell	Cat# BE0241
*Klebsiella pneumoniae* CIP52.145 purified capsule polysaccharide	Bengoechea laboratory, Queen’s University Belfast (UK)	N/A
Recombinant mouse IFN-β	PBL Assay Science	Cat# 12401-1
pCMV-pro-IL1β-C-Flag	Luke O'Neill laboratory, Trinity College Dublin (Ireland)	RRID: Addgene_75128
pcDNA3-N-Flag-Caspase-1	Luke O'Neill laboratory, Trinity College Dublin (Ireland)	RRID: Addgene_75128
pcDNA3-N-Flag-ASC1	Luke O'Neill laboratory, Trinity College Dublin (Ireland)	RRID: Addgene_75134
Mouse SARM1 in pdlNotInPkMCSR	Andrew Bowie laboratory, Trinity College Dublin (Ireland)	N/A
Mouse SARM1 TIR in pdlNotInPkMCSR	Andrew Bowie laboratory, Trinity College Dublin (Ireland)	N/A
Mouse SARM1 ΔTIR in pdlNotInPkMCSR	Andrew Bowie laboratory, Trinity College Dublin (Ireland)	N/A
HA-MyD88	S. Miggin, Institute of Immunology, National University of Ireland, Maynooth (Ireland)	N/A
HA-TRIF	C. Basler laboratory, Mount Sinai School of Medicine, New York (USA)	N/A
pCMV-HA-human AIM2	Andrew Bowie laboratory, Trinity College Dublin (Ireland)	PMID: 35013241
pdlNotInPkMCSR	Andrew Bowie laboratory, Trinity College Dublin (Ireland)	PMID 23431397

**Critical commercial assays**		

ELISA TNF-α	PeproTech	Cat# 900-K54
ELISA IL-1β	PeproTech	Cat# 900-K47
ELISA IL10	PeproTech	Cat# 900-K53
ELISA IP10	Peprotech	Cat# 250-16
QUANTI-Blue	InvivoGen	Cat# rep-qbs3
Cresyl violet acetate salt	Sigma-Aldrich	Cat# C5042
Neutral Red	Sigma-Aldrich	Cat# N7005
ELISA IL-1b	R&D Systems	Cat# SMLB00C
Maxpar X8 Antibody Labelling Kit	Fluidigm	Cat# SKU 201149A

**Experimental models: Cell lines**		

iBMDM *Il-10*^−/−^	Bengoechea laboratory, Queen’s University Belfast (UK)	PMID: 30745327
iBMDM *irf3*^−/−^	Bengoechea laboratory, Queen’s University Belfast (UK)	PMID: 29112952
iBMDM *ifnar1*^*−/−*^	Bengoechea laboratory, Queen’s University Belfast (UK)	N/A
iBMDM *sarm1*^−/−^	Bengoechea laboratory, Queen’s University Belfast (UK)	N/A
iBMDM *Sarm1*^*em1.1Tf*^	Bengoechea laboratory, Queen’s University Belfast (UK)	N/A
iBMDM *Sarm1*^*FLAG*^	Bengoechea laboratory, Queen’s University Belfast (UK)	N/A
iBMDM *aim2*^−/−^	Bengoechea laboratory, Queen’s University Belfast (UK)	N/A
iBMDM *nlrp3*^−/−^	Bengoechea laboratory, Queen’s University Belfast (UK)	N/A
iBMDM *casp-1*^−/−^	Bengoechea laboratory, Queen’s University Belfast (UK)	N/A
iBMDM *asc*^−/−^	Bengoechea laboratory, Queen’s University Belfast (UK)	N/A
iBMDM *gsdmd*^−/−^	Bengoechea laboratory, Queen’s Universit Belfast (UK)	N/A

**Experimental models: Organisms/strains**		

C57BL/6 mice	Charles River	Cat# 027
*sarm1*^*−/−*^, B6.129X1-Sarm1tm1Aidi/J	The Jackson Laboratory, bred at Qeen’s University Belfast (UK)	Cat# JAX stock #018069
*Sarm1*^*em1.1Tft*^	Bowie laboratory, Trinity College Dublin (Ireland)	PMID: 34793837
*Sarm1*^Flag^, *Sarm1*^em2(FLAG−Strep)Tftc^	Bowie laboratory, Trinity College Dublin (Ireland)	PMID: 34793837

**Software and algorithms**		

ImageJ	NIH	https://imagej.nih.gov/ij/index.html
FlowJo Software	FlowJo	N/A
GraphPad Prism	GraphPad Software, Inc	N/A
CyTOF software	Fluidgm	N/A
RStudio software	https://www.rstudio.com/	N/A
Cytofkit package	https://github.com/JinmiaoChenLab/cytofkit	N/A

### Resource availability

#### Lead contact

Further information and requests for resources and reagents should be directed to and will be fulfilled by the lead contact, Jose Bengoechea (j.bengoechea@qub.ac.uk).

#### Materials availability

Cell lines generated in this study are available from the lead contact upon request.

### Experimental model and subject details

C57BL/6 mice (Charles River), and *sarm1*^*−/−*^, B6.129X1-Sarm1tm1Aidi/J mice were obtained from The Jackson Laboratory, and bred at Queen’s University Belfast. *Sarm1*^*em1.1Tf*^ and *Sarm1*^*FLAG*^ were generated and bred at Trinity Biomedical Sciences Institute (TBSI), Trinity College Dublin, by the Bowie laboratory and they have been recently described (Doran et al., 2021). Mice were age and sex-matched and used between 8-12 weeks of age. The experiments involving mice were approved by the Queen’s University Belfast’s Ethics Committee and conducted in accordance with the UK Home Office regulations (project licences PPL2778 and PPL2910) issued by the UK Home Office. Animals were randomized for interventions but researches processing the samples and analysing the data were aware which intervention group corresponded to which cohort of animals.

### Method details

#### Bacterial strains and growth conditions

Kp52145 is a clinical isolate (serotype O1:K2) previously described (Lery et al., 2014; Nassif et al., 1989). The *cps* mutant strain, 52145-Δ*manC*, the mutant lacking the LPS O-polysaccharide, 52145-Δ*glf*, and the double mutant lacking the CPS and the LPS O-polysaccharide, 52145-Δ*wca*_*k2*_- Δ*glf*, are isogenic strains of Kp52145 and they have been described previously (Kidd et al., 2017; Sa-Pessoa et al., 2020). Strain 52145-Δ*glf* expresses similar levels of CPS than the wild-type strain (Sa-Pessoa et al., 2020). KP35 and NJST258_2 are two *K. pneumoniae* strains of the ST258 clonal group previously described (Ahn et al., 2016; Deleo et al., 2014).

Bacteria were grown in 5 mL Luria-Bertani (LB) medium at 37°C on an orbital shaker (180 rpm), and where appropriate, antibiotics were added to the growth medium at the following concentration: carbenicllin, 50 μg/mL; chloramphenicol, 25 μg/mL.

#### Mammalian cells and cell culture

iBMDMs cells from wild-type (WT), *tlr4*^−/−^, *myd88*^−/−^, and *tram*^*−/−*^*trif*^*−/−*^ mice on a C57BL/6 background were obtained from BEI Resources (NIAID, NIH) (repository numbers NR-9456, NR-9458, NR-15633, and NR-9568, respectively). *Il-10*^−/−^ and *irf3*^−/−^ iBMDMs were described previously (Bartholomew et al., 2019; Ivin et al., 2017). Additional iBMDMs were generated as previously described (Sa-Pessoa et al., 2020). Briefly, tibias and femurs from C57BL/6, *ifnar1*^*−/−*^, *sarm1*^−/−^, *Sarm1*^*em1.1Tf*^, *Sarm1*^*FLAG*^*, aim2*^−/−^, *nlrp3*^−/−^, *casp-1*^−/−^, *asc*^−/−^, and *gsdmd*^−/−^ were removed using sterile techniques, and the bone marrow was flushed with fresh medium. To obtain macrophages, cells were plated in Dulbecco’s modified Eagle’s medium (DMEM) supplemented with 20% filtered L929 cell supernatant (a source of macrophage colony-stimulating factor) and maintained at 37°C in a humidified atmosphere of 5% CO2. Medium was replaced with fresh supplemental medium after 1 day. Immortalization of BMDMs was performed after 5 days by exposing them h to the J2 CRE virus (carrying v-myc and v-Raf/v-Mil oncogenes, kindly donated by Avinash R. Shenoy, Imperial College London) for 24. This step was repeated 2 days later (day 7), followed by continuous culture in DMEM supplemented with 20% (vol/vol) filtered L929 cell supernatant for 4 to 6 weeks. The presence of a homogeneous population of macrophages was assessed by flow cytometry using antibodies for CD11b (clone M1/70; catalog number 17-0112-82; eBioscience) and CD11c (clone N418; catalog number 48-0114-82; eBioscience). Retroviral transduction of SARM1 in *sarm1*^−/−^ cells was done as previously described (Carty et al., 2006, 2019).

iBMDMs and BMDMs were grown in DMEM (catalog number 41965; Gibco) supplemented with heat-inactivated fetal calf serum, 100 U/mL penicillin, and 0.1 mg/mL streptomycin (Gibco) at 37°C in a humidified 5% CO2 incubator. Cells were routinely tested for *Mycoplasma* contamination. Cells were seeded a density of 2 × 10^4^ cells/well in 24-well plates, 5 × 10^5^ cells/well in 12-well plates, and 2 × 10^6^ cells/well in 6-well plates.

#### Infection conditions

Overnight bacterial cultures were refreshed 1/10 into a new tube containing 4.5 mL of fresh LB. After 2.5 h at 37°C, bacteria were pelleted (2500× g, 20 min, 22°C), resuspended in PBS and adjusted to an optical density of 1.0 at 600 nm (5 × 10^8^ CFU/mL). Infections were performed using a multiplicity of infection (MOI) of 100 bacteria per cell in a 1 mL volume. Synchronization of the infection was performed by centrifugation (200 × g for 5 min). For incubation times longer than 30 min, cells were washed and 1 mL of fresh medium containing gentamycin (100 μg/mL) was added to the wells to kill extracellular bacteria. Medium containing gentamycin was kept until the end of the experiment. Infections were performed one day after seeding the cells in the same medium used to maintain the cell line without antibiotics. Infected cells were incubated at 37°C in a humidified 5% CO2 incubator.

#### siRNA experiments

For transfection of siRNAs, 2 × 10^4^ iBMDMs (6-well plates) were transfected in suspension with 20 nM siRNA using Lipofectamine RNAiMAX (Invitrogen) in 200 μL Opti-MEM I (ThermoFisher). AllStars negative-control siRNA (Qiagen) or ON-TARGET plus SMART pool siRNA targeting AIM2 (no. L-044968-01-0020; Dharmacon) and SARM1 (no. L-041633-01-0005; Dharmacon) were used to transfect cells. The macrophages were infected 16 h post transfection. Efficiency of transfection was confirmed by RT-qPCR analysis of duplicate samples from three independent transfections by normalizing to the hypoxanthine phosphoribosyltransferase 1 (*hprt*) gene and comparing gene expression in the knockdown sample with the AllStars negative control. Primers are listed in Table S3.

#### Inhibitors, recombinant cytokines, blocking antibodies, and purified CPS

The NLRP3 inhibitor MCC950 ([vehicle solution DMSO], 10 μM CAS 256373-96-3 – Calbiochem, Sigma-Aldrich), and the caspase 1 inhibitor YVAD ([vehicle solution DMSO], 10 μM CAS 256373-96 Sigma-Aldrich) were added 2 h before infection to the cells. Recombinant mouse IL-10 ([vehicle solution water] 1 ng/mL, Biolegend) was added overnight before infection. The p38 inhibitor SB203580 ([vehicle solution DMSO], 10 μM, Sigma-Aldrich) was added 2 h before infection. The mouse anti-IFNAR1 receptor antibody (clone MAR1-5A3 [vehicle solution water] 5 ng/mL, BioXcell) was added overnight before infection. All these reagents were kept for the duration of the experiment. Purified CPS was obtained and characterized as previously described by our laboratory (Regueiro et al., 2009).

#### RNA isolation and RT-qPCR

Infections were performed in 6-well plates. Cells were washed three times with pre-warmed sterile PBS, and total RNA was extracted from the cells in 1 mL of TRIzol reagent (Ambion) according to the manufacturer’s instructions. Extracted RNA was treated with DNase I (Roche) and precipitated with sodium acetate (Ambion) and ethanol. RNA was quantified using a Nanovue Plus spectrophotometer (GE Healthcare Life Sciences). cDNA was generated by retrotranscription of 1 g of total RNA using M-MLV reverse transcriptase (Invitrogen) and random primers (Invitrogen). Two duplicates were generated from each sample. Ten nanograms of cDNA were used as a template in a 5- L reaction mixture from a KAPA SYBR FAST qPCR kit (Kapa Biosystems). Primers used are listed in Table S3. RT-qPCR was performed using a Rotor-Gene Q (Qiagen) with the following thermocycling conditions: 95°C for 3 min for hot-start polymerase activation, followed by 40 cycles of 95°C for 5 s and 60°C for 20 s. Fluorescence of SYBR green dye was measured at 510 nm. Relative quantities of mRNAs were obtained using the ΔΔC_T_ method by using hypoxanthine phosphoribosyltransferase 1 (*hprt*) gene normalization.

#### Immunoblots

Macrophages were seeded in 6-well plates for 24 h before infection. Cell lysates were prepared in lysis buffer (1× SDS Sample Buffer, 62.5 mM Tris-HCl pH 6.8, 2% w/v SDS, 10% glycerol, 50 mM DTT, 0.01% w/v bromophenol blue). Proteins were resolved on 8, 10 or 12% SDS-PAGE gels and electroblotted onto nitrocellulose membranes. Membranes were blocked with 3% (wt/vol) bovine serum albumin in TBS-Tween (TBST), and specific antibodies were used to detect protein using chemiluminescence reagents and a G:BOX Chemi XRQ chemiluminescence imager (Syngene).

The following antibodies were used: anti-IL-1β (anti-goat, 1:1,000; # AF-401-NA, R&D Systems), anti-caspase-1 (anti-rabbit, 1:1,000; #24232, Cell Signaling), anti-AIM2 (anti-rabbit, 1:1,000; sc-515895, Santa Cruz), anti-NLRP3 (anti-mouse, 1:1,000; #15101, Cell Signaling), anti-Gasdermin-D (anti-rabbit, 1:1,000; #93709, Cell Signaling), anti-Viperin (anti-rabbit, 1:1,000 # NBP2-03971, Novus Biologicals), anti-ISG15 (1:1,000; #2743, Cell Signaling), anti-phospho-STAT3 (anti-rabbit, 1:1,000; #9145, Cell Signaling), anti-IκBα (anti-rabbit, 1:1,000; #4814, Cell Signaling), anti-phospho-IκBα (Ser32) (anti-goat, 1:1,000; #9246, Cell Signaling), anti-phospho-AKT1/2/3 (Ser 473) (anti-rabbit, 1:1,000; sc-33437, Santa Cruz), anti-phospho-IKKα/β (Ser176/180)(16A6) (anti-rabbit, 1:1,000; #2697,Cell Signaling), anti-phospho-IRF3 (Ser 396) (anti-rabbit, 1:1,000; #4947, Cell Signaling), anti-phospho-p-TBK-1/NAK (Ser172) (D52C2) (anti-rabbit, 1:1,000; #5483, Cell Signaling), anti-phospho-JNK (anti-rabbit, 1:1,000; #9251S, Cell Signaling), anti-phospho-ERK (anti-rabbit, 1:1,000; #9101, Cell Signaling), anti-phospho-p38 MAPK (Thr180/Tyr182) (D3F9) (anti-rabbit, 1:1,000; #4511, Cell Signaling), anti-SARM1 (anti-chicken, 1:70; generated by Icosagen by immunizing chicken with the TIR domain of human SARM1), anti-Flag M2 (1 μg, Sigma-Aldrich F3165), anti-HA (1:1,000, Santa Cruz sc-805). Immunoreactive bands were visualized by incubation with HRP-conjugated IgG Secondary antibody (anti-goat, 1:5,000; # HAF017, R&D Systems, goat anti-rabbit, 1:5,000; #170-6515, Bio Rad, goat anti-mouse, 1:5,000; #6516, Bio-Rad). To ensure that equal amounts of proteins were loaded, blots were re-probed with α-tubulin (1:3,000; #T9026, Sigma- Aldrich) or β-actin (anti-mouse, 1:1,000; sc-130065, Santa Cruz). To detect multiple proteins, membranes were re-probed after stripping of previously used antibodies using a pH 2.2 glycine-HCl/SDS buffer.

#### Densitometry analysis

MAPKs bands were quantified using ImageJ and normalized to the loading control. Graphs represent fold change compared to non infected cells set to 1.

#### Processing cell free supernatants for inflammasome studies

iBMDMs were seeded in 6 wells plates and were infected 24 h later. At the indicated time points, the plates were centrifuged at 200xg for 5 min at room temperature, and the supernatants were transferred to microcentrifuge tubes and placed on ice. The cells were lysed in 80 μL of Laemmeli buffer with β-mercaptoethanol (1 in 19 ratio), collected in a microcentrifuge tube and stored at −20°C. The supernatants were processed by adding 9 μL of StrataClean Resin, hydroxylated silica particles (Cat. 400714) per 1 mL of supernatant. The samples were homogenized in vortex for 1 min, and were centrifuged at 9000 × g for 2 min. The supernatant was discarded, and the pellets were suspended in 40 μL of Laemmli buffer and transferred to filtered columns within collection tubes. The columns were centrifuged at 8,000 × g at RT for 1 min, and the eluate collected. The samples were boiled for 5 min in heat block at 95°C and loaded for western blot analysis.

#### Enzyme-linked immunosorbent assay (ELISA), and cytokine measurement

Infections were performed in 12-well plates. Supernatants from infected cells were collected at the indicated time points in the figure legends, and spun down at 12,000 × g for 5 min to remove any debris. TNF-α (#900-K54), IL-1β (#900-K47), IL-10 (#900-K53) and IP-10 (CXCL10) (#250-16) in the supernatants were quantified using ABTS ELISA Development Kit (PeproTech) according to the manufacturer’s instructions. All experiments were performed in duplicate, and three independent experiments were conducted.

For quantification of type I IFN (INF-α/β) in the supernatants of iBMDMs, cells were infected for 16 h, and supernatants were collected. Murine type I IFNs were detected using B16-Blue IFN-α/β reporter cells (InvivoGen) which carry an SEAP reporter gene under the control of the IFN-α/β-inducible ISG54 promoter and that have an inactivation of the IFN-γ receptor. Supernatants from iBMDM cells were incubated with the reporter cell line, and levels of SEAP in the supernatants were determined using the detection medium QUANTI-Blue (InvivoGen) after 24 h as per the manufacturer’s instructions using recombinant mouse IFN-β (PBL Assay Science, catalogue number 12401-1) as a standard. Experiments were run in duplicates and repeated at least three times. Results are expressed as OD at 655 nm.

#### Detection of ASC specks formation by flow cytometry

To detect ASC speck formation by flow cytometry, we adapted the protocol described by Sester and colleagues (Sester et al., 2015). Cells were harvested from 6-wells plates with ice-cold PBS, centrifuged at 1,000 × g for 5 min, and resuspended in 1 mL ice-cold PBS. Samples were then fixed by the drop wise addition of 4 mL ice-cold molecular grade ethanol while vortexing. After 15 min, cells were pelleted by centrifugation at 600 × g for 10 min, supernatants gently removed and pellets suspended in 250 μL ASC speck buffer (ASB, PBS/0.1% sodium azide, 0.1% BSA, 1.5% FCS) containing 1 μL Fc block anti-CD16/CD32 (2.4G2, BD Biosciences) for 20 min. To stain ASC specks, 0.2 μL anti-ASC (Cat# sc-22514R, Santa Cruz) in 50 μL ASB buffer were added to the samples, and incubated for 90 min at room temperature. The cells were washed with 1 mL ASB, and the re suspended in 50 μL ASB containing 0.1 μL Alexa 488 goat anti-rabbit IgG (H+L) (Molecular Probes). After 45 min, cells were washed with 1 mL ASB, and re suspended in 500 μL ASB. Samples were processed on a BD FACS Canto and analyzed using FlowJo X (Tree Star) software and graphical representation.

#### AIM2 reconstitution in HEK cells

HEK293T cells were seeded at 2 × 10^5^ cells/well in 24-well plates and incubated overnight. The cells were transfected using Lipofectamine 2000 with plasmids expressing pro-IL-b-FLAG (50 ng), pro-Caspase-1-FLAG (10 ng), ASC-FLAG (1 ng), HA-AIM2 (50 ng) and 10, 50 or 100 ng of pdlNotInPkMCSR FLAG SARM1, FLAG SARM1 TIR, FLAG SARM1 ΔTIR or pdlNotInPkMCSR empty vector control. Medium was replaced 24 h after transfection and supernatants were collected 16 h after media change. Quantification of secreted murine IL-1β was performed using ELISA (R&D Systems). Cells were lysed with RIPA buffer and subjected to immunoblotting by using anti-HA or anti-FLAG antibodies for the detection of AIM2 and SARM/SARM TIR/SARM ΔTIR expression.

#### Coimmunoprecipiration analysis

iBMDMs were seeded onto 6-wells plates (8 × 10^5^ cells/well). Cells were transfected the following day with 1 μg of MyD88-HA or TRIF-HA plasmids (Carty et al., 2006) diluted in 200 μL of opti-MEM (Gibco) using 6 μL of Lipofectamine 2000 (Invitrogen). Transfected cells were infected 20 h post transfection at a MOI of 100. After 1 h of contact, media was replaced by media containing gentamicin (100 μg/mL), and cells were collected at 3 h and lysed in RIPA buffer containing: 50 mM Tris-HCl, pH 7.2, 0.15 M NaCl, 0.1% SDS, 1% Sodium Deoxycholate, 1% Triton X-100 and proteinase inhibitors: 1 mM PMSF and halt protease inhibitor cocktail (ThermoFisher Scientific, catalogue number 78430). The whole cell lysates were centrifuged at 10,000 ×g for 20 min at 4°C. The supernatants were transferred to a new tube and the pellets were kept to probe the input. Whole cell lysates were incubated with 1 μg FLAG (Sigma-Aldrich, F3165) or normal mouse IgG (Santa Cruz, c-2025) antibodies for 2 h at 4°C in a rotary wheel mixer. Protein A/G Plus agarose suspension (Santa Cruz # sc-2003) was added to the whole cells lysates suspension and incubated at 4°C on a rotary mixer overnight. The suspension was centrifuged at 1,000 ×g for 4 min at 4°C and the supernatant was aspirated and discarded. Pellets were washed 2 times with RIPA buffer, suspended in 40 μL of 2 × electrophoresis sample buffer (Laemmli buffer) and boiled for 5 min at 95°C.

#### Cell viability

To assess cell viability we adapted the protocol of Repetto and colleagues (Repetto et al., 2008). Cells were seeded in 96 well plate to a density of 50,000–54,000 cells per well 16 h pre infection. Cells were infected at a multiplicity of infection of 100 to 1 in a final volume of 190 μL antibiotic free DMEM tissue culture medium supplemented with 10% FCS. Synchronization of the infection was performed by centrifugation (200 × g for 5 min). After 60 min, cells were washed once with PBS, and 180 μL of fresh medium containing gentamycin (100 μg/mL) were added to the wells to kill extracellular bacteria. Medium containing gentamycin was kept until the end of the experiment. Cells were incubated for 23 h. After, they were washed twice with PBS, and incubated with 100 μL of freshly prepared neutral red medium (final concentration 40 μg/mL (w/v) neutral red (Sigma-Aldrich) in tissue culture medium) for 2 h. Cells were washed once with PBS, and the uptaken neutral red by the cells was released by incubation of the cells with 150 μL destaining solution (50% ethanol 96%; 49% deionised water, 1% glacial acetic acid) at room temperature for 15 min with agitation. The released neutral red was quantified by determining the OD_540_ in a plate reader (POLARstar Omega). Cell viability of infected cells was compared to that of non- infected cells set to 100%. Experiments were carried out in triplicate on three independent occasions.

#### Adhesion, phagocytosis and intracellular survival

iBMDMs were seeded in 12-well plates approximately 16 h before infection. Infections were performed as previously described. To enumerate the number of bacteria adhered to macrophages, after 30 min of contact cells were washed twice with PBS, and they were lysed in 300 μL of 0.1% (wt/vol) saponin in PBS for 5 min at 37°C. Serial dilutions were plated in LB and the following day bacterial CFUs were counted. Results are expressed as CFU per ml. To determine the number of bacteria phagocytosed by the cells, after 30 min of contact, cells were washed once with PBS and fresh medium containing gentamycin (100 μg/mL) was added to the wells. After 30 min, cells were washed three times with PBS, and lysed with saponin. Samples were serially diluted in PBS and plated in PBS. After 24 h incubation at 37°C, CFUs were counted and results expressed as CFUs per ml. To assess intracellular survival, 4 h after the addition of gentamycin, cells were washed three times with PBS and lysed with saponin. Serial dilutions were plated on LB to quantify the number of intracellular bacteria. Results are expressed as % of survival (CFUs at 4 h versus 1 h in *sarm1*^−/−^ cells normalized to the results obtained in wild-type macrophages set to 100%). All experiments were carried out with triplicate samples on at least five independent occasions.

#### Assessment of the colocalization of the KCV with cellular markers

The protocol was adapted from (Cano et al., 2015). Briefly, wild-type and *sarm1*^−/−^ iBMDMs (2 × 10^4^ per well) were grown on 13 mm circular coverslips in 24-well plates and were infected with Kp52145 harbouring pFPV25.1Cm (March et al., 2013). After 30 min of contact the coverslips were washed with PBS and gentamycin (100 μg/mL in DMEM medium) was added to kill extracellular bacteria.

##### Staining of lysosomes

Cresyl violet acetate salt (Sigma-Aldrich) was used to label lysosomes (Ostrowski et al., 2016). Cresyl violet in fresh medium (5μM) was added to the cells 12 min before fixing the cells. The residual fluid marker was removed by washing the cells three times with PBS, followed by fixation (4% paraformaldehyde in PBS pH 7.4 for 20 min at room temperature). Coverslips were mounted with ProLong Gold antifade mountant (Invitrogen). Coverslips were visualised on the Leica SP8 Confocal microscope within 24 h after fixing. To determine the percentage of bacteria that co-localized with cresyl violet, bacteria located inside a minimum of 100 infected cells were analysed in each experiment. Experiments were carried out in duplicate in three independent occasions.

##### Rab14 staining

At the indicated time points post infection, coverslips were washed with PBS and permeabilized with 0.1% (w/v) saponin (Sigma) in PBS for 30 min. Coverslips were then incubated for 120 min with anti-Rab14 (4 μg/mL in 0.1% (v/v) horse serum (Gibco), 0.1% (w/v) saponin in PBS; clone D-5, murine IgG1, sc-271401, Santa Cruz), washed with PBS, followed by a 45 min incubation with anti-mouse IgG H&L labelled with AlexaFluor 647 (10 μg/mL in 0.1% (v/v) horse serum (Gibco), 0.1% (w/v) saponin in PBS, polyclonal, donkey IgG, ab150111, Abcam). Coverslips were washed with PBS, and then incubated with anti-Lamp1 (1 μg/mL in 0.1% (v/v) horse serum (Gibco), 0.1% (w/v) saponin in PBS, clone 1D4B, rat IgG2a, sc-19992, Santa Cruz) for 20 min, washed with PBS, and incubated for 20 min with anti-rat IgG H&L labelled with AlexaFluor 568 (10 μg/mL in 0.1% (v/v) horse serum (Gibco), 0.1% (w/v) saponin in PBS, polyclonal, goat IgG, A11077, Life Technologies). Coverslips were mounted in microscope slides with ProLong Gold antifade mountant (Invitrogen), and visualised on a TCS-SP5 inverted microscope (Leica Biosystems). To determine the percentage of the Lamp1 positive KCV that co-localized with Rab14, KCVs of at least 100 infected cells from three independent experiments were analysed.

#### Intranasal murine infection model

Infections were performed as previously described (Ivin et al., 2017). Briefly, 8- to 12-week-old C57BL/6 mice (Charles River), *sarm1*^*−/−*^, B6.129X1-Sarm1tm1Aidi/J (The Jackson Laboratory, and bred at Queen’s University Belfast), *Sarm1*^*em1.1Tft*^ (Doran et al., 2021) of both sexes were infected intranasally with ∼3 × 10^5^ Kp52145 in 30 μL PBS. Non-infected mice were mock infected with 30 μL sterile PBS. The number of mice per group are indicated in the figure legends. 24 h post infection, mice were euthanized using a Schedule 1 method according to UK Home Office approved protocols. For those mice used for mass cytometry analysis, 16 h post infection, they were dosed intraperitoneally with 500 μg of monensin (Sigma-Aldrich) for intracellular cytokine staining.

Left lung samples from infected and uninfected control mice were immersed in 1 mL of RNA stabilisation solution (50% [w/v] ammonium sulphate, 2.9% [v/v] 0.5 M ethylenediaminetetraacetic acid, 1.8% [v/v] 1 M sodium citrate) on ice and then stored at 4°C for at least 24 h prior to RNA extraction. Samples were homogenized in 1 mL ice-cold TRIzol (Ambion) using a VDI 12 tissue homogenizer (VWR). RNA was extracted according to the manufacturer’s instructions extraction, and cDNA was generated by retrotranscription of 1 μg of total RNA using M-MLV reverse transcriptase (Invitrogen) and random primers (Invitrogen). RT-qPCR analysis was undertaken using the KAPA SYBR FAST qPCR Kit, oligonucleotide primers as described in the *in vitro* protocol, and Rotor-Gene Q (Qiagen). Thermal cycling conditions were as follows: 95°C for 3 min for enzyme activation, 40 cycles of denaturation at 95°C for 10 s and annealing at 60°C for 20 s. Each cDNA sample was tested in duplicate, and relative mRNA quantity was determined by the comparative threshold cycle (ΔΔC_T_) method using hypoxanthine phosphoribosyltransferase 1 (m*hprt*) gene normalisation.

Right lung, spleen and liver samples from infected mice were immersed in 1 mL sterile PBS on ice and processed for quantitative bacterial culture immediately. Samples were homogenised with a Precellys Evolution tissue homogenizer (Bertin Instruments), using 1.4 mm ceramic (zirconium oxide) beads at 4,500 rpm for 7 cycles of 10 s, with a 10-s pause between each cycle. Homogenates were serially diluted in sterile PBS and plated onto *Salmonella-Shigella* agar (Sigma-Aldrich), and the colonies were enumerated after overnight incubation at 37°C. Data were expressed as CFUs per gr of tissue.

#### Mass cytometry

##### Generation of metal-labelled antibodies

Carrier protein and glycerol-free antibodies were labelled with lanthanide isotopes using Maxpar X8 Antibody Labelling Kits (Fluidigm) according to the manufacturer’s instructions. Briefly, X8 polymer was loaded with the lanthanide isotype in L-buffer, and the metal-loaded polymer purified and washed in C-buffer using an Amicon Ultra-0.5 centrifugal filter unit with 3 kDa cutoff (Millipore-Sigma). At the same time, the antibody was reduced with 4 mM tris(2-carboxyethyl)phosphine hydrochloride (TCEP) solution in R-buffer, and purified in C-buffer, using an Amicon Ultra-0.5 centrifugal filter unit with 50 kDa cut-off (Millipore-Sigma).

Both the lanthanide-loaded polymer and the partially reduced antibody were mixed and incubated at 37°C for 90 min. Once the incubation was completed, the conjugated antibody was washed several times with W-buffer using an Amicon Ultra-0.5 centrifugal filter unit with 50 kDa cut-off (Millipore-Sigma), and quantified using a NanoDrop spectrophotometer (280 nm). The antibody was finally resuspended in antibody stabilizer PBS supplemented with 0.05% sodium azide at a final concentration of 0.5 mg/mL and stored at 4°C.

##### Mass cytometry staining and acquisition

Mice lungs were aseptically collected in PBS and homogenized with a handheld homogenizer. Single-cell suspensions were obtained by flushing the samples through 70 μM strainer, incubated with nuclease (Pierce). Red blood cells were lysed with ACK buffer, and samples stained, according to manufacturer’s instructions. Briefly, cell suspensions were first incubated with 1 μM of 103Rh for live/dead discrimination, and later with cell surface metal-labelled antibodies, prepared in Maxpar Cell Staining Buffer (CSB; Fluidigm), for 30 min at room temperature. Cells were washed with CSB, fixed and permeabilized with Maxpar Fix I buffer (Fluidigm) for 10 min at room temperature, washed with 2 volumes ofMaxpar Perm-S buffer (Fluidigm), and incubated with metal-labelled antibodies for intracellular markers, prepared in Maxpar Perm-S buffer, for 30 min at room temperature. The list of antibodies used is shown in Table S1. Finally, samples were washed with CSB, incubated 10 min at room temperature with a 2% paraformaldehyde solution, washed once more with CSB, and left at 4°C in Maxpar Fix and Perm buffer (Fluidigm) with 125 nM Cell-ID™ Intercalator Ir (Fluidigm) until acquisition. Samples were acquired between 12 and 48 h after staining. Right before acquisition, cells were washed with CSB, followed by Maxpar Cell Acquisition Solution (CAS; Fluidigm). Cells were resuspended in CAS with 1 mM EDTA to a final concentration of 1 × 10^6^ cells/mL, flushed through a 35 μM strainer, and supplemented with 1/10 v/v EQ Four Element Calibration Beads (Fluidigm). Mass cytometry was performed using a Helios CyTOF instrument (Fluidigm) operated with software v7.0.8493. The CyTOF instrument was started, tuned, and cleaned according to the manufacturer’s protocol, and samples acquired with an injection speed of 30 μL/min.

##### Mass cytometry data analysis

Data was exported as flow-cytometry FCS file format, and pre-processed with CyTOF software (v6.7.1014; Fluidigm) for normalization. Processed files were uploaded to the Cytobank platform (https://www.cytobank.org/) for initial gating (Gaussian parameters and cells/beads, live/dead and singlets/doublets discriminations). CD45^+^ populations were gated and exported in FCS file format an analysed with RStudio software (https://www.rstudio.com/) and cytofkit package (https://github.com/JinmiaoChenLab/cytofkit) for Phenograph clustering using the following parameters: 10.000 cells/sample, cytofAsinh as transformation Method, Phenograph as cluster method, k equal to 30 as Rphenograph, tsne as visualization method, a seed of 42.

### Quantification and statistical analysis

Statistical analyses were performed using one-way analysis of variance (ANOVA) with Bonferroni corrections, the one-tailed t test, or, when the requirements were not met, the Mann-Whitney U test. p values of <0.05 were considered statistically significant. Normality and equal variance assumptions were tested with the Kolmogorov-Smirnov test and the Brown-Forsythe test, respectively. All analyses were performed using GraphPad Prism for Windows (version 9.1.0) software.

## Data Availability

• Data reported in this paper will be shared by the lead contact upon request. • This paper does not report original code. • Any additional information required to reanalyze the data reported in this work paper is available from the lead contact upon request.

## Supplemental information

Document S1. Figures S1–S9 and Tables S1–S3

Document S2. Article plus supplemental information
