# Supplementary material for: Klebsiella pneumoniae hijacks the Toll-IL-1R protein SARM1 in a type I IFN-dependent manner to antagonize host immunity
Source: Cell Rep. 2022 Aug 9;40(6):111167. doi: 10.1016/j.celrep.2022.111167 (PMC9638020; doi:10.1016/j.celrep.2022.111167)
Supplement: Document S1. Figures S1–S9 and Tables S1–S3 [file mmc1.pdf]

Cell Reports, Volume *40*

Supplemental information

Klebsiella pneumoniae hijacks the Toll-IL-1R protein SARM1 in a type I IFN-dependent manner to antagonize host immunity

Claudia Feriotti, Joana Sá-Pessoa, Ricardo Calderón-González, Lili Gu, Brenda Morris, Ryoichi Sugisawa, Jose L. Insua, Michael Carty, Amy Dumigan, Rebecca J. Ingram, Adrien Kissenpfening, Andrew G. Bowie, and José A. Bengoechea

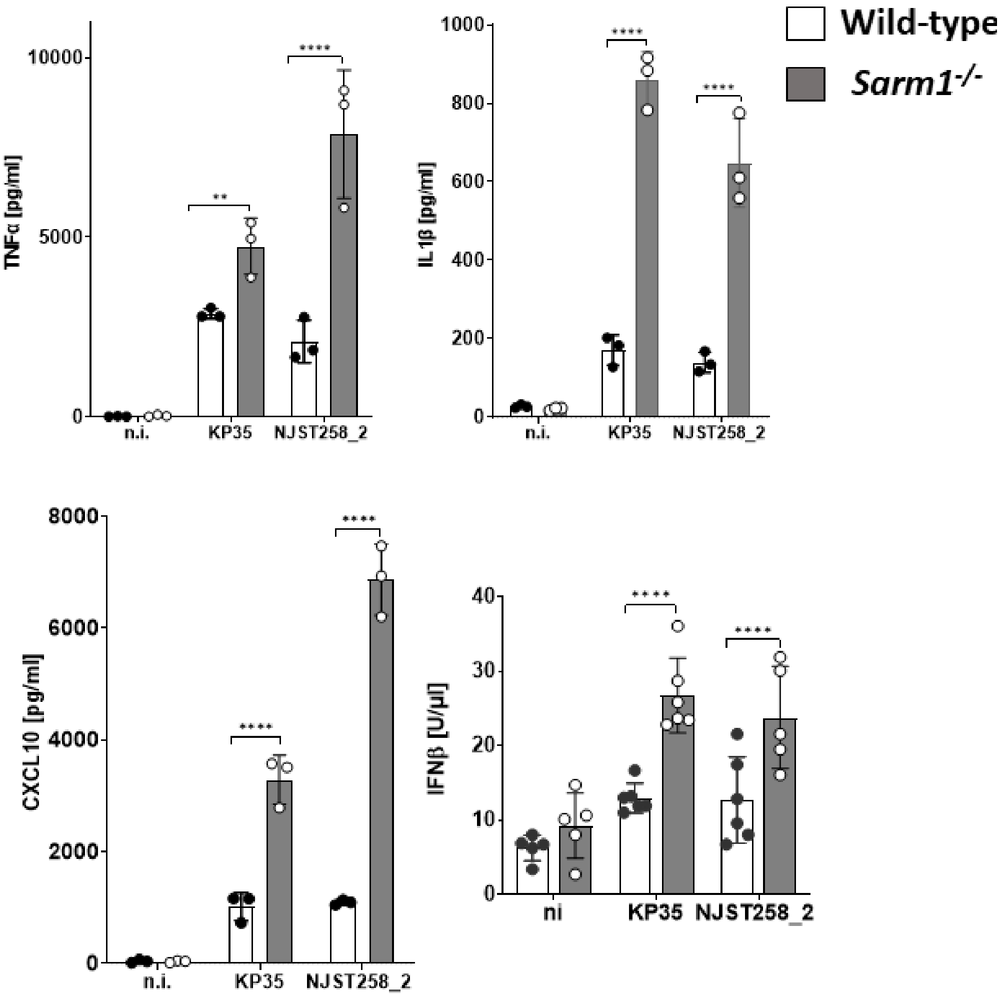

Figure S1 related to Figure 1. SARM1 negatively regulates inflammation induced by carbapenem *K. pneumoniae* strains.

ELISA of TNFα, IL1β, CXCL10 secreted by wild-type (WT) and *sarm1*$^{-/-}$ macrophages following infection with KP35 and NJST258_2 for 16 h. Type I IFN levels determined in the supernatants of macrophages 16 h post infection.

In all infections, after 1 h contact, the medium was replaced with medium containing gentamicin (100 µg/ml) to kill extracellular bacteria.

In all panels, values are presented as the mean ± SD of three independent experiments measured in duplicate. ****$P \leq 0.0001$; **$P \leq 0.01$; for the indicated comparisons using unpaired t test.

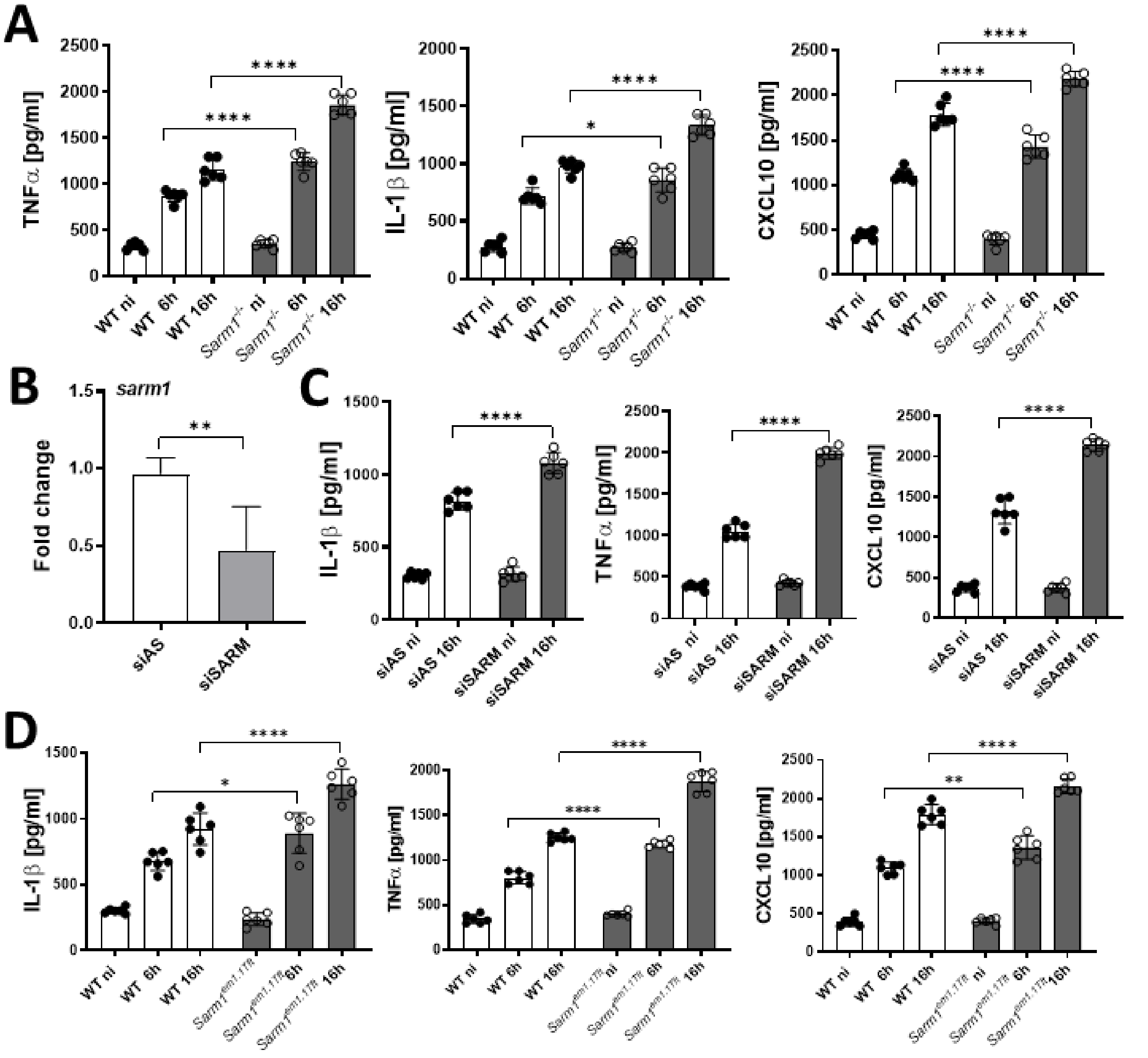

Figure S2 related to Figure 1. SARM1 negatively regulates *K. pneumoniae*-induced inflammation.

A. ELISA of TNFα, IL1β, CXCL10 secreted by wild-type (WT) and *sarm1*$^{-/-}$ BMDMs non-infected (ni) or infected with Kp52145 for 6 and 16 h.

B. Efficiency of transfection of SARM siRNA (siSARM) in wild-type iBMDMs. mRNA levels were assessed 16 h post transfection as fold change against control non-silencing agents AllStars (siAS).

C. ELISA of IL1β, TNFα, and CXCL10 secreted by wild-type (WT) macrophages transfected with All Stars siRNA control (siAS), or SARM1 siRNA (siSARM) non-infected (ni) or infected with Kp52145 for 16 h.

D. ELISA of IL1β, TNFα, and CXCL10 secreted by wild-type (WT) and *Sarm1*$^{em1.1Tft}$ macrophages non-infected (ni) or infected with Kp52145 for 6 and 16 h.

In all infections, after 1 h contact, the medium was replaced with medium containing gentamicin (100 μg/ml) to kill extracellular bacteria.

In panels A, C, and D, values are presented as the mean ± SD of three independent experiments measured in duplicate. ****P ≤ 0.0001; **P≤ 0.01; *P ≤ 0.05; for the indicated comparisons using one way-ANOVA with Bonferroni contrast for multiple comparisons test. In panel B, **P≤ 0.01 using unpaired t test.

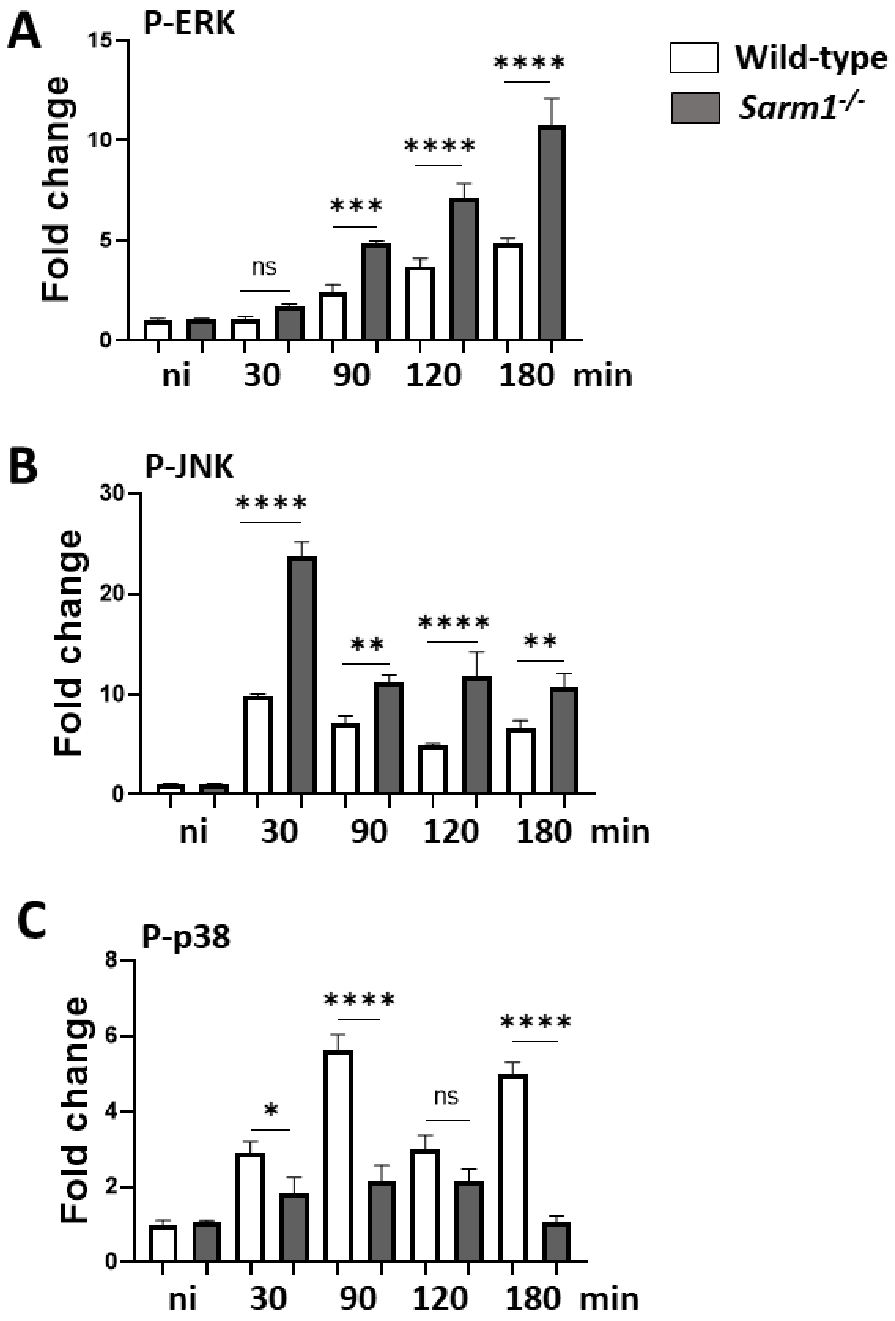

Figure S3 related to Figure 1. Densitometry analysis.

Quantification of the relative intensity of phosphorylated bands of MAPKs ERK (A), JNK (B), and p38 (C) normalized to tubulin following infection of wild-type macrophages with Kp52145 for the indicated time points. The value of non-infected cells was set to 1.

Values are presented as the mean ± SD of three independent blots. ****P ≤ 0.0001; ***P ≤ 0.001; ** P ≤ 0.01; *, P ≤ 0.05; ns, P > 0.05 for the indicated comparisons using one way-ANOVA with Bonferroni contrast for multiple comparisons test.

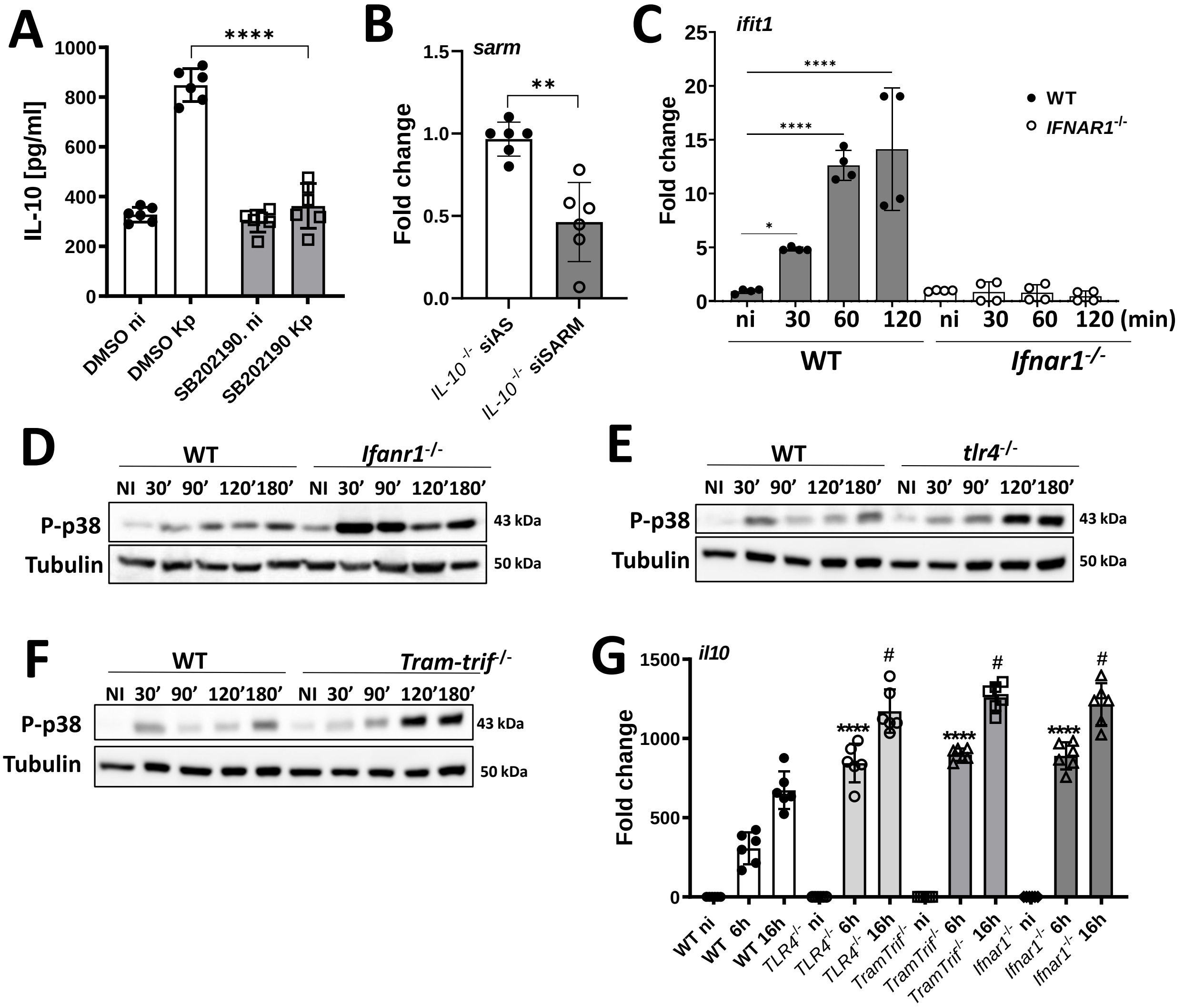

Figure S4 related to Figure 2. *K. pneumoniae* induction of IL10 is controlled by p38 and it is negatively regulated by type I IFN.

A. ELISA of IL10 secreted by wild-type macrophages non-infected (ni) or infected with Kp52145 (Kp) 16 h. Cells were treated with the p38 inhibitor SB202190 or DMSO vehicle control.

B. Efficiency of transfection of SARM1 siRNA (siSARM) in *il10*$^{-/-}$ macrophages. mRNA levels were assessed 16 h post transfection as fold change against control non-silencing agents AllStars (siAS).

C. *ifit1* mRNA levels were assessed by qPCR, in wild-type (WT), and *ifnar1*$^{-/-}$ macrophages non-infected (ni) or infected with Kp52145 for the indicated time points.

D. Immunoblot analysis of phosphorylated p38 (P-p38), and tubulin levels in lysates of wild-type (WT) and *ifnar1*$^{-/-}$ macrophages non-infected (NI) or infected with Kp52145 for the indicated time points.

E. Immunoblot analysis of phosphorylated p38 (P-p38), and tubulin levels in lysates of wild-type (WT) and *tlr4*$^{-/-}$ macrophages non-infected (NI) or infected with Kp52145 for the indicated time points.

F. Immunoblot analysis of phosphorylated p38 (P-p38), and tubulin levels in lysates of wild-type (WT) and *tram*$^{-/-}$*trif*$^{-/-}$ macrophages non-infected (NI) or infected with Kp52145 for the indicated time points.

G. *il10* mRNA levels were assessed by qPCR, in wild-type (WT), *tlr4*$^{-/-}$, *tram*$^{-/-}$*trif*$^{-/-}$, and *ifnar1*$^{-/-}$ macrophages non-infected (ni) or infected with Kp52145 for 6 and 16 h.

In all infections, after 1 h contact, the medium was replaced with medium containing gentamicin (100 µg/ml) to kill extracellular bacteria.

In panels A, B and G, values are presented as the mean ± SD of three independent experiments measured in duplicate. In panel C, values are presented as the mean ± SD of two independent experiments measured in duplicate. In panels D, E and F the images are representative of three independent experiments. In panel A and C, ****$P \leq 0.0001$; * $P \leq 0.05$ for the indicated comparisons using one way-ANOVA with Bonferroni contrast for multiple comparisons test. In panel B, **$P \leq 0.01$ using unpaired t test. In panel G, ****$P \leq 0.0001$ for the comparison between infected knock-out and wild-type cells for 6 h; # $P \leq 0.0001$ for the comparison between infected knock-out and wild-type cells for 16 h using one way-ANOVA with Bonferroni contrast for multiple comparisons test.

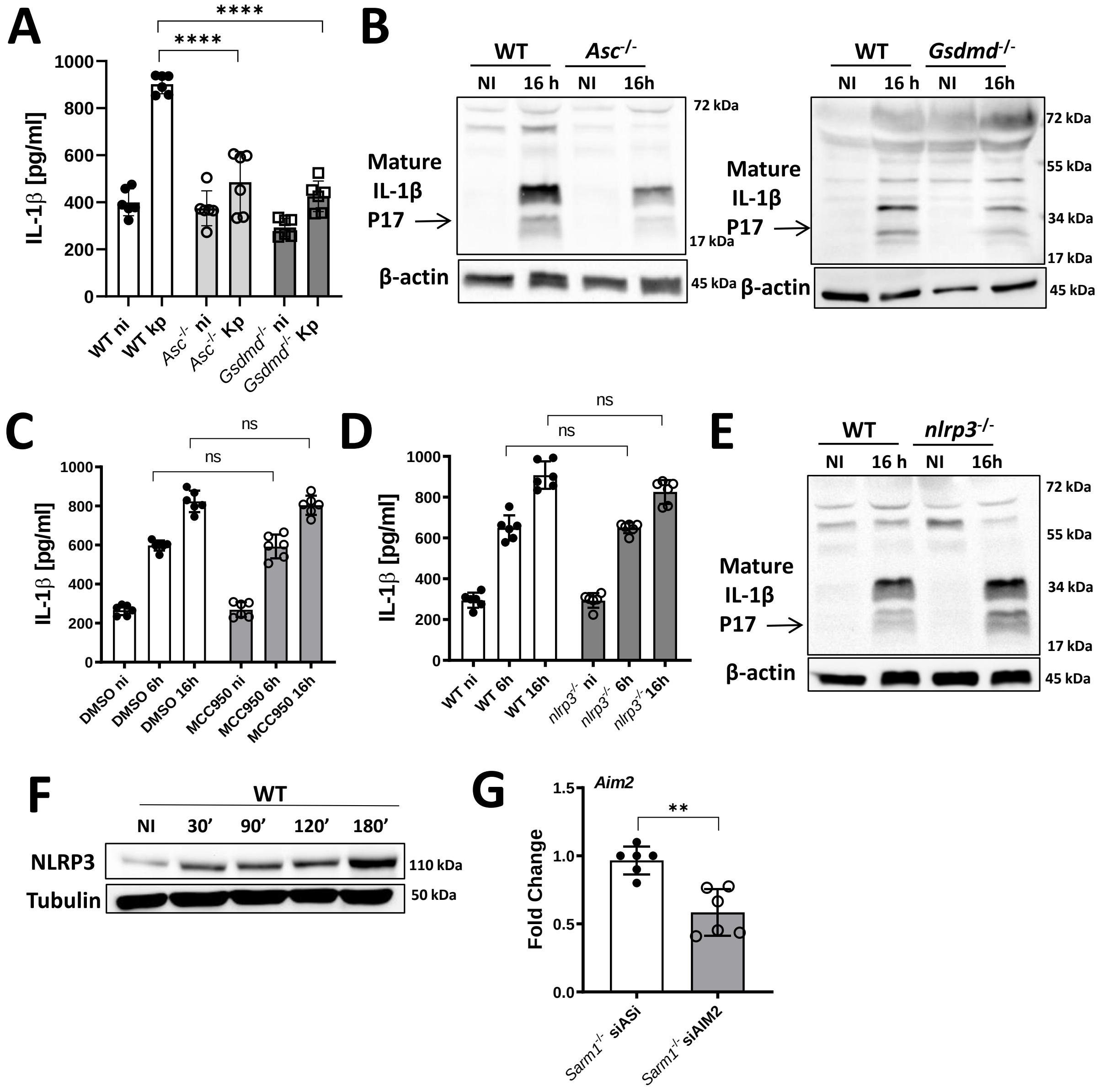

Figure S5 related to Figure 3. *K. pneumoniae* does not activate NLRP3 inflammasome.

A. ELISA of IL1β secreted by wild-type (WT), *asc*$^{-/-}$, and *gsmd*$^{-/-}$ macrophages non-infected (ni) or infected with Kp52145 (Kp) for 16 h.

B. Immunoblot analysis of processed pro-IL1β, and β-actin levels in lysates of wild-type macrophages (WT) and *asc*$^{-/-}$ and *gsmd*$^{-/-}$ macrophages non-infected or infected with Kp52145 for 16h.

C. ELISA of IL1β secreted by wild-type (WT) macrophages non-infected (ni) or infected with Kp52145 (Kp) for 6 and 16 h. Cells were treated with the NLRP3 inhibitor MC950 or DMSO vehicle control.

D. ELISA of IL1β secreted by wild-type (WT) and *nlrp3*$^{-/-}$ macrophages non-infected (ni) or infected with Kp52145 for 6 and 16 h.

E. Immunoblot analysis of processed pro-IL1β, and β-actin levels in lysates of wild-type macrophages (WT) and *nlrp3*$^{-/-}$ macrophages non-infected or infected with Kp52145 for 16h.

F. Immunoblot analysis of NLRP3 and tubulin levels in lysates of wild-type macrophages (WT) and *nlrp3*$^{-/-}$ macrophages non-infected (NI) or infected with Kp52145 for the indicated time points.

G. Efficiency of transfection of AIM2 siRNA (siAIM2) in sarm1$^{-/-}$ macrophages. mRNA levels were assessed 16 h post transfection as fold change against control non-silencing agents AllStars (siAS).

In all infections, after 1 h contact, the medium was replaced with medium containing gentamicin (100 μg/ml) to kill extracellular bacteria.

In panels A, C and D values are presented as the mean ± SD of three independent experiments measured in duplicate. ****P ≤ 0.0001; ns, P > 0.05 for the indicated comparisons using one way-ANOVA with Bonferroni contrast for multiple comparisons test. In panel G, values are presented as the mean ± SD of three independent experiments measured in duplicate. **P≤ 0.01 using unpaired t test. In panels B, E and F, images are representative of three independent experiments.

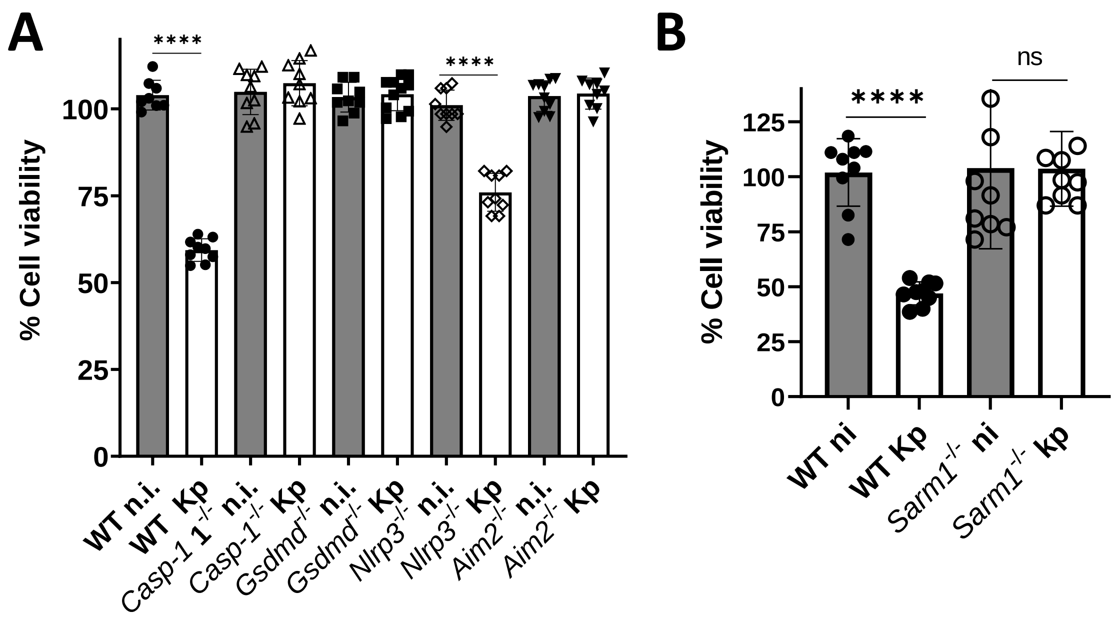

Figure S6 related to Figure 3. Cell viability.

Cell viability was assessed by quantifying the binding of the supravital dye neutral red to the lysosomes.

A. Percentage of cell viability following infection with Kp52145 for 23 of wild-type (WT), caspase $1^{-/-}$, *gsmd*$^{-/-}$, *nlrp3*$^{-/-}$ and *aim2*$^{-/-}$ macrophages. The value of non-infected wild-type cells was set to 100%.

B. Percentage of cell viability following infection with Kp52145 for 23 of wild-type (WT), and *sarm1*$^{-/-}$ macrophages. The value of non-infected wild-type cells was set to 100%.

In all infections, after 1 h contact, the medium was replaced with medium containing gentamicin (100 µg/ml) to kill extracellular bacteria.

Values are presented as the mean ± SD of three independent experiments measured in triplicate. ****$P \leq 0.0001$; ns, $P > 0.05$ for the indicated comparisons using unpaired t test.

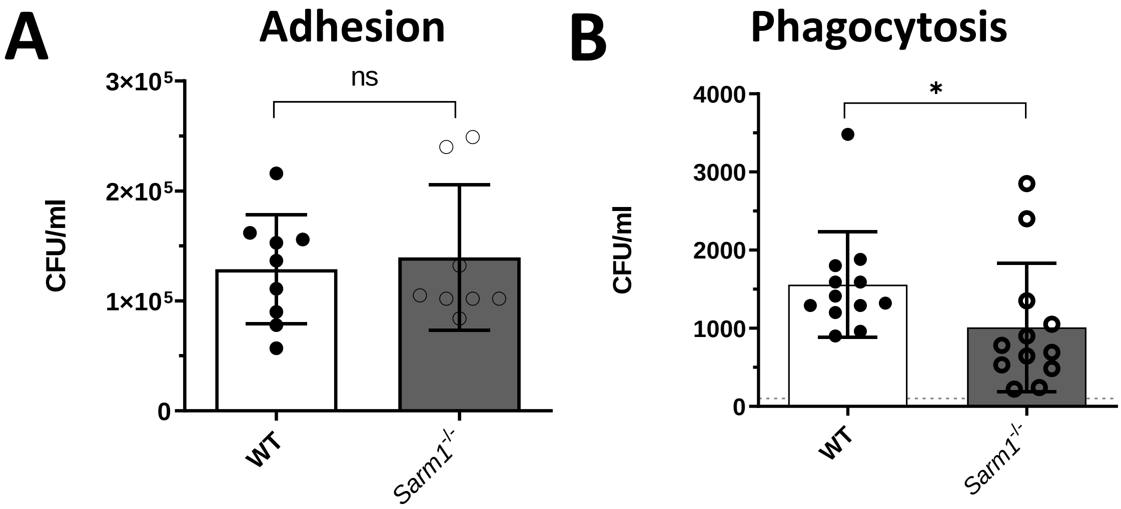

Figure S7 related to Figure 6. Adhesion and phagocytosis of *K. pneumoniae* by *sarm1*$^{-/-}$ macrophages.

A. Adhesion in wild-type (WT) and *sarm1*$^{-/-}$ macrophages. Cells were infected with Kp52145 for 30 min, wells were washed and bacteria were quantified by lysis, serial dilution and viable counting on LB agar plates.

B. Phagocytosis of Kp52145 by wild-type (WT) and *sarm1*$^{-/-}$ macrophages. Cells were infected for 30 min, wells were washed, and it was added medium containing gentamicin (100 µg/ml) to kill extracellular bacteria. After 30 min, cells were washed and bacteria were quantified by lysis, serial dilution and viable counting on LB agar plates.

In panels A and B, values are presented as the mean ± SD of three independent experiments measured in triplicate. * $P \leq 0.05$; ns, $P > 0.05$ for the indicated comparisons using unpaired t test.

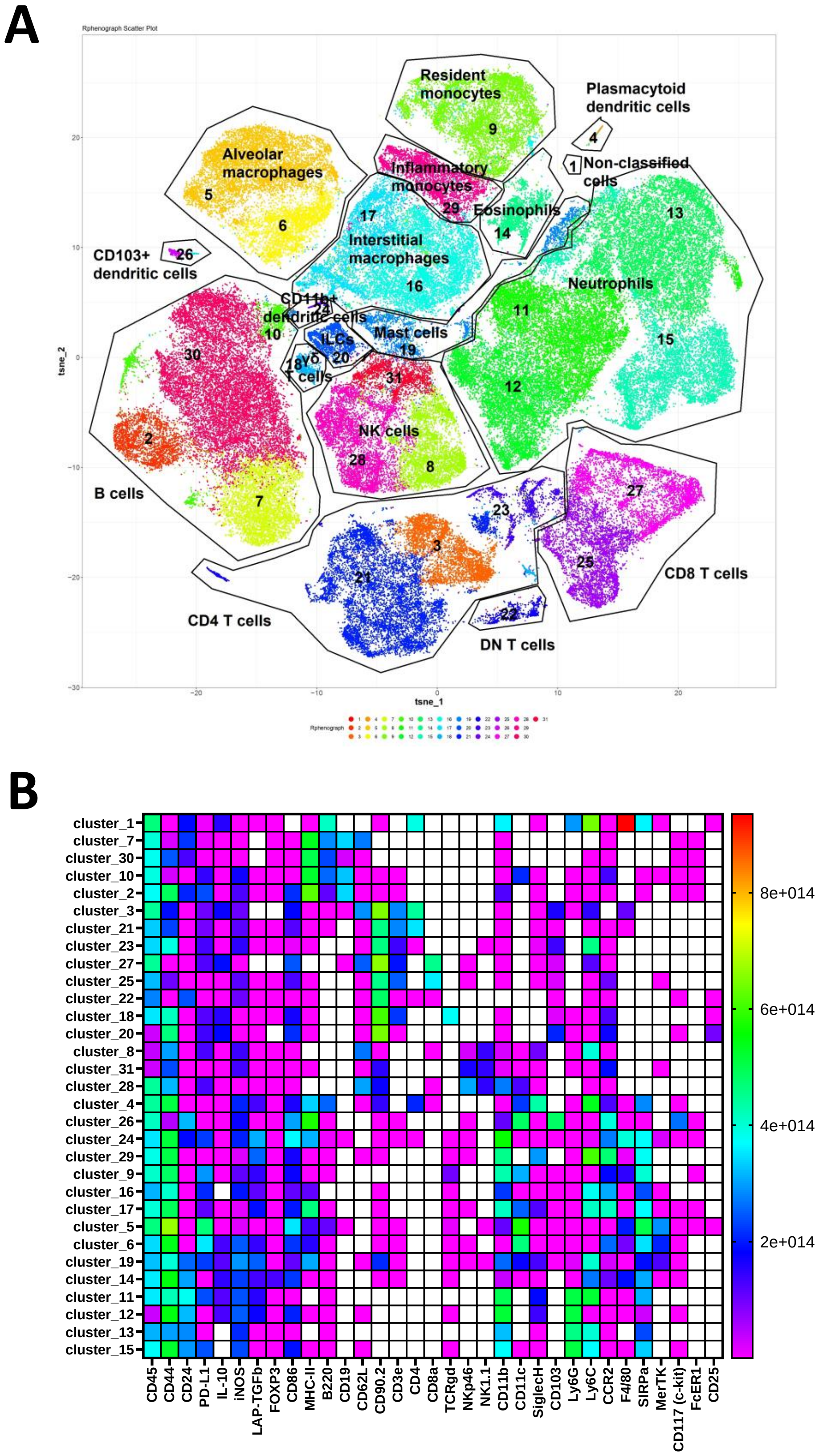
A
Rphenograph Scatter Plot
Resident monocytes
Plasmacytoid dendritic cells
Alveolar macrophages
Inflammatory monocytes
Non-classified cells
Eosinophils
Interstitial macrophages
CD103+ dendritic cells
Neutrophils
CD11b+ dendritic cells
ILCs
Mast cells
γδ T cells
NK cells
B cells
CD8 T cells
CD4 T cells
DN T cells
tsne_1
tsne_2
Rphenograph
B
cluster_1
cluster_7
cluster_30
cluster_10
cluster_2
cluster_3
cluster_21
cluster_23
cluster_27
cluster_25
cluster_22
cluster_18
cluster_20
cluster_8
cluster_31
cluster_28
cluster_4
cluster_26
cluster_24
cluster_29
cluster_9
cluster_16
cluster_17
cluster_5
cluster_6
cluster_19
cluster_14
cluster_11
cluster_12
cluster_13
cluster_15
CD45
CD44
CD24
PD-L1
IL-10
iNOS
LAP-TGFb
FOXP3
CD86
MHC-II
B220
CD19
CD62L
CD90.2
CD3e
CD4
CD8a
TCRgd
NKp46
NK1.1
CD11b
CD11c
SiglecH
CD103
Ly6G
Ly6C
CCR2
F4/80
SIRPa
MerTK
CD117 (c-kit)
FcER1
CD25
8e+014
6e+014
4e+014
2e+014

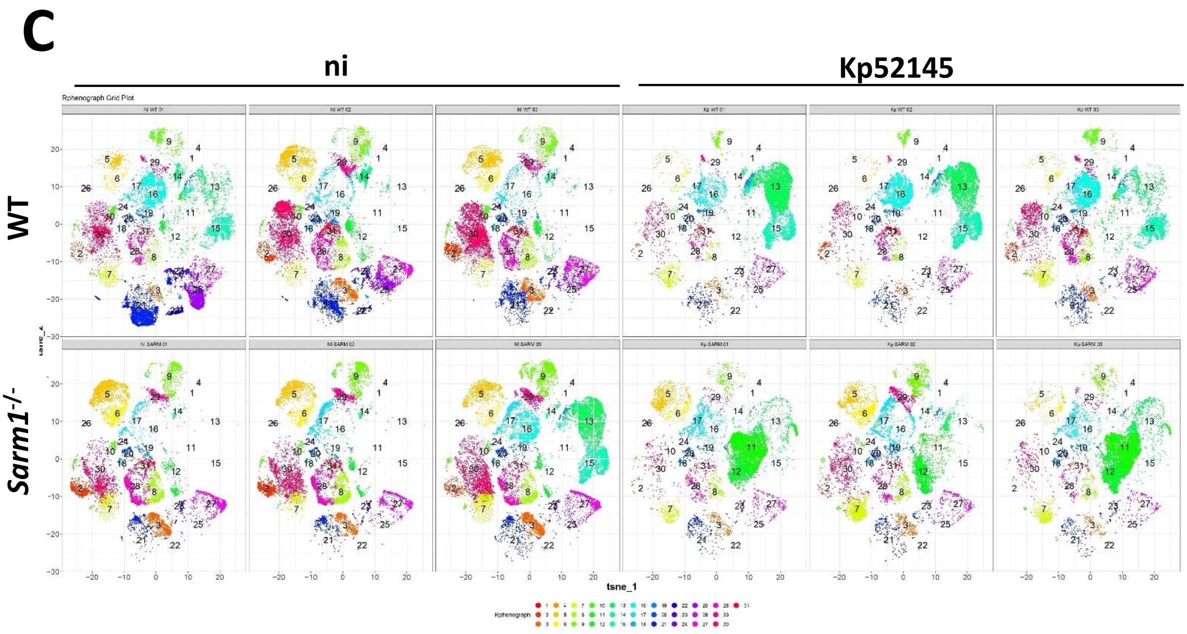

Figure S8 related to Figure 7. Description of mouse immune populations following *K. pneumoniae* infection.

A. PhenoGraph cluster analysis of immune populations in the lungs wild-type (WT), and *sarm1*$^{-/-}$ mice non-infected (ni) or infected intranasally with Kp52145 for 24. Graphs shows the combine results of all groups.

B. Heat map showing relative signal intensities of the indicated markers on the clusters identified in panel A. The heat map is coloured based on signal intensity of the indicated markers. Results are based on data from three mice per group.

C. PhenoGraph cluster analysis of immune populations in the lungs wild-type (WT), and *sarm1*$^{-/-}$ mice non-infected (ni) or infected intranasally with Kp52145 for 24. Each graph represents an individual mouse.

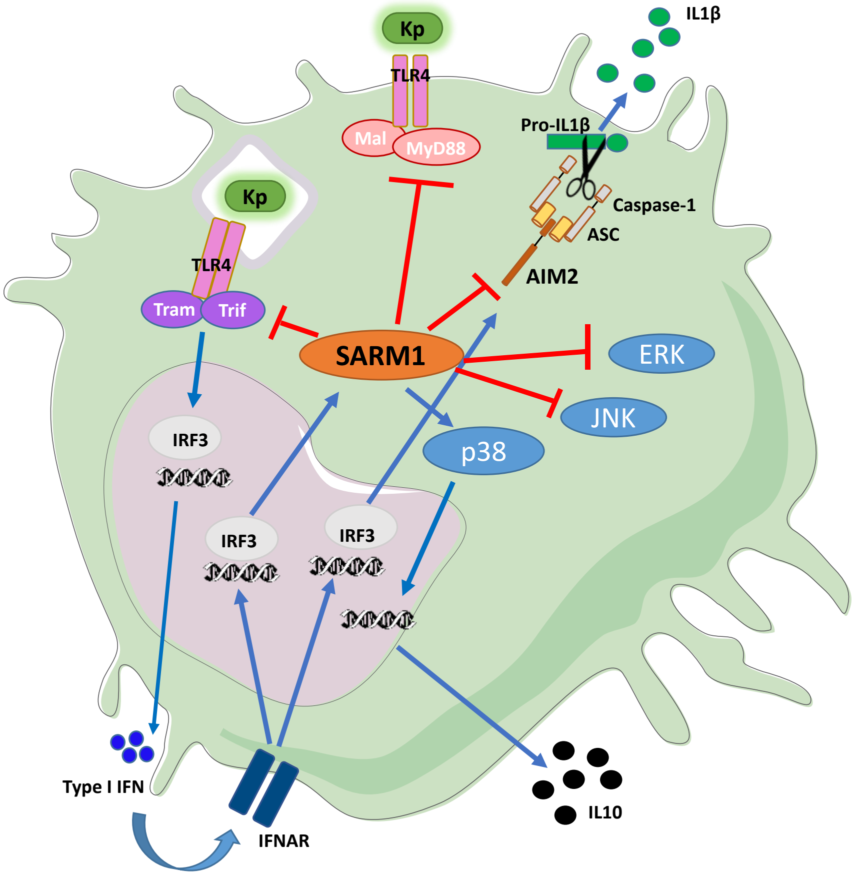

Figure S9. ***K. pneumoniae*** **exploits the immunomodulatory properties of SARM1 to antagonize cell intrinsic immunity.**

Kp52145 activates the signalling pathway TLR4-TRAM-TRIF-IRF3 to induce the production of type I IFN, which signals through the IFNAR1 receptor (Ivin et al., 2017). Type I IFN stimulates the transcription of SARM1, and AIM2 via IRF3. SARM1 negatively regulates MyD88 and TRIF-governed inflammatory responses, the activation of the MAP kinases ERK and JNK, and the AIM2 inflammasome. In contrast, SARM1 is required for the activation of the MAP kinase p38, which controls the production of IL10. Kp52145 exploits IL10 to control inflammation. Absence of SARM1 impairs the intracellular survival of Kp52145, and *sarm1*$^{-/-}$ mice do control Kp52145 infection. Collectively, our findings illustrate the crucial role of SARM1 in *K. pneumoniae* immune evasion strategies.

Table S1. Antibodies used to characterize immune populations by mass cytometry.

Marker	Metal isotope	Clone	Reference
Ly6G	141Pr	1A8	Fluidigm 3141008B
SIRPa	143Nd	P84	BD 552371*
B220	144Nd	RA3-6B2	Fluidigm 3144011B
CD4	145Nd	RM4-5	Fluidigm 3145002B
F4/80	146Nd	BM8	Fluidigm 3146008B
CD45	147Sm	30-F11	Fluidigm 3147003C
CD11b	148Nd	M1/70	Fluidigm 3148003C
CD19	149Sm	6D5	Fluidigm 3149002B
CD24	150Nd	M1/69	Fluidigm 3150009B
CD25	151Eu	3C7	Fluidigm 3151007B
CD3e	152Sm	145-2C11	Fluidigm 3152004B
PD-L1	153Eu	10F.9G2	Fluidigm 3153016B
CD103	155Gd	2E7	BioLegend 121402*
CD90.2	156Gd	30-H12	Fluidigm 3156006B
IL-10	158Gd	JES5-16E3	Fluidigm 3158002C
TCRgd	159Tb	GL3	Fluidigm 3159012B
CD62L	160Gd	MEL-14	Fluidigm 3160008C
iNOS	161Dy	CXNFT	Fluidigm 3161011B
Ly6C	162Dy	HK1.4	Fluidigm 3162014B
SiglecH	163Dy	551	BioLegend 129602*
LAP/TGFb	164Dy	TW7-16B4	Fluidigm 3164014B
FOXP3	165Ho	FJK-16S	Fluidigm 3165024A
CCR2	166Er	475301R	R&D MAB55381R*
CD335/NKp46	167Er	29A1.4	Fluidigm 3167008B
CD8a	168Er	53-6.7	Fluidigm 3168003B
MerTK	169Tm	*Polyclonal*	R&D AF591*
CD161/NK1.1	170Er	PK136	Fluidigm 3170002C
CD44	171Yb	IM7	Fluidigm 3171003C
CD86	172Yb	GL-1	Fluidigm 3172016B
CD117/c-kit	173Yb	2B8	Fluidigm 3173004B
MHC-II	174Yb	M5/114.15.2	Fluidigm 3174003B
FcER1	176Yb	MAR-1	Fluidigm 3176006B
CD11c	209Bi	N418	Fluidigm 3209005B

*****Antibodies conjugated to the indicated metal isotope using Maxpar X8 Antibody Labelling Kit.

Table S2. Immune populations identified by mass cytometry.

Population	Subpopulation	Cluster	Markers
B cells	Naïve B cells	7	B220^{+}CD19^{+}MHC-II^{+}CD62L^{high}
	Mature B cells	30	B220^{+}CD19^{+}MHC-II^{+}CD62L^{low}
	CD11c^{+} B cells	10	B220^{+}CD19^{+}MHC-II^{+}CD62L^{low}CD11c^{+}
	Plasma cells	2	B220^{low}CD19^{+}MHC-II^{+}
CD4 T cells	Naïve CD4 T cells	3	CD90.2^{+}CD3^{+}CD4^{+}CD62L^{high}
	Mature CD4 T cells	21	CD90.2^{+}CD3^{+}CD4^{+}CD62L^{low}
	Ly6C^{+} mature CD4 T cells	23	CD90.2^{+}CD3^{+}CD4^{+}CD62L^{low}Ly6C^{high}
CD8 T cells	Naïve CD8 T cells	27	CD90.2^{+}CD3^{+}CD8^{+}CD62L^{high}
	Mature CD8 T cells	25	CD90.2^{+}CD3^{+}CD8^{+}CD62L^{low}
Double negative T cells		22	CD90.2^{+}CD3^{+}CD4$^{-/low}$CD8$^{-/low}$
Gamma delta T cells		18	CD90.2^{+}CD3^{+}TCRgd^{+}
Innate lymphoid cells		20	CD90.2^{+}CD3$^{-/low}$
NK cells	CD90.2^{-} NK cells	8	CD90.2$^{-/low}$NK1.1^{+}NKp46^{+}
	CD90.2^{+}CD62L^{-} NK cells	31	CD90.2^{+}NK1.1^{+}NKp46^{+}CD62L$^{-/low}$
	CD90.2^{+}CD62L^{+} NK cells	28	CD90.2^{+}NK1.1^{+}NKp46^{+}CD62L^{+}
Plasmacytoid dendritic cells		4	MHC-II^{+}CD11c^{+}B220^{+}SiglecH^{+}F4/80$^{-/low}$
Myeloid dendritic cells	CD103+ dendritic cells	26	MHC-II^{+}CD11c^{+}CD11b^{low}CD103^{+}F4/80$^{-/low}$
	CD11b+ dendritic cells	24	MHC-II^{+}CD11c^{low}CD11b^{+}CD103^{-}F4/80^{+}
Inflammatory monocytes		29	MHC-II^{-}Ly6G^{-}Ly6C^{+}CD11b^{+}CD11c^{-}CCR2^{high}
Resident monocytes		9	MHC-II^{-}Ly6G^{-}Ly6C^{-}CD11b^{+}CD11c^{+}CCR2^{+}
Interstitial macrophages	CD11c^{-} interstitial macrophages	16	MHC-II^{+}Ly6G^{-}Ly6C^{+}CD11b^{+}CD11c$^{-/low}$
	CD11c^{+} interstitial macrophages	17	MHC-II^{+}Ly6G^{-}Ly6C^{+}CD11b^{+}CD11c^{+}
Alveolar macrophages	CCR2^{-} alveolar macrophages	5	MHC-II^{+}Ly6G^{-}Ly6C^{-}CD11b^{low}CD11c^{+}CCR2^{-}
	CCR2^{+} alveolar macrophages	6	MHC-II^{+}Ly6G^{-}Ly6C^{-}CD11b^{-}CD11c^{+}CCR2^{+}
Mast cells/basophils		19	MHC-II^{+}Ly6G^{-}Ly6C^{+}F4/80$^{-/low}$CD90.2^{+}CD11b^{+} CD11c^{+}
Eosinophils		14	MHC-II^{-}Ly6G^{-}Ly6C^{+}F4/80^{+}
Neutrophils	SiglecH^{high}PD-L1^{+} neutrophils	11	MHC-II^{-}Ly6G^{+}Ly6C^{+}F4/80$^{-/low}$SiglecH^{high}PD-L1^{high}
	SiglecH^{high}PD-L1^{-} neutrophils	12	MHC-II^{-}Ly6G^{+}Ly6C^{+}F4/80$^{-/low}$SiglecH^{high}PD-L1^{low}
	SiglecH^{low}PD-L1^{-} neutrophils	13	MHC-II^{-}Ly6G^{+}Ly6C^{+}F4/80$^{-/low}$SiglecH^{low}PD-L1^{low}
	SiglecH^{low}PD-L1^{+} neutrophils	15	MHC-II^{-}Ly6G^{+}Ly6C^{+}F4/80$^{-/low}$SiglecH^{low}PD-L1^{high}

Table S3. Primers used in this study.

Name	Sequence (5'-3')
mSARM Forward	GGT GCA CAA GGA GAT TGT GAC
mSARM Reverse	CAT GGG ACC ATT TGA TGC CGT T
mIL1B-F1	AGA TGA AGG GCT GCT TCC AAA
mIL1B-R1	AAT GGG AAC GTC ACA GAC CA
mTNFa-F1	TTC TGT CTA CTG AAC TTC GGG GTG ATC GGT CC
mTNFa-R1	GTA TGA GAT AGC AAA TCG GCT GAC GGT GTG GG
mIFNb-F	ATG GTG GTC CGA GCA GAG AT
mIFNb-R	CCA CCA CTC ATT CTG AGG
mCXCL10-F1	AGT GCT GCC GTC ATT TTC TG
mCXCL10-R1	ATT CTC ACT GGC CCG TCA T
mISG15-F	GGG GCC ACA GCA ACA TCT AT
mISG15-R	CGC TGG GAC ACC TTC TTC TT
m.Mx1_F1	GAC TAC CAC TGA GAT GAC CCA GC
m.Mx1_R1	ATT TCC TCC CCA AAT GTT TTC A
mIFIT1-F	CAG GTT TCT GAG GAG TTC TG
mIFIT1-R	TGA AGC AGA TTC TCC ATG AC
mIL10_F1	GGA CTT TAA GGG TTA CTT GGG TTG CC
mIL10_R1	CAT GTA TGC TTC TAT GCA GTT GAT GA
mIL12_p40_F1	GGA AGC ACG GCA GCA GAA TA
mIL12_p40_R1	AAC TTG AGG GAG AAG TAG GAA TGG
AIM2_Fwd	GTT GAA TCT AAC CAC GAA GTC C
AIM2_Rvr	CTA CAA GGT CCA GAT TTC AAC TG
